# Polymer-tetrodotoxin conjugates to induce prolonged duration local anesthesia with minimal toxicity

**DOI:** 10.1038/s41467-019-10296-9

**Published:** 2019-06-12

**Authors:** Chao Zhao, Andong Liu, Claudia M. Santamaria, Andre Shomorony, Tianjiao Ji, Tuo Wei, Akiva Gordon, Hannes Elofsson, Manisha Mehta, Rong Yang, Daniel S. Kohane

**Affiliations:** Laboratory for Biomaterials and Drug Delivery, Division of Critical Care Medicine, Boston Children’s Hospital, Harvard Medical School, 300 Longwood Avenue, Boston, MA 02115 USA

**Keywords:** Pharmaceutics, Medical research, Drug delivery

## Abstract

There is clinical and scientific interest in developing local anesthetics with prolonged durations of effect from single injections. The need for such is highlighted by the current opioid epidemic. Site 1 sodium channel blockers such as tetrodotoxin (TTX) are extremely potent, and can provide very long nerve blocks but the duration is limited by the associated systemic toxicity. Here we report a system where slow release of TTX conjugated to a biocompatible and biodegradable polymer, poly(triol dicarboxylic acid)-co-poly(ethylene glycol) (TDP), is achieved by hydrolysis of ester linkages. Nerve block by the released TTX is enhanced by administration in a carrier with chemical permeation enhancer (CPE) properties. TTX release can be adjusted by tuning the hydrophilicity of the TDP polymer backbone. In vivo, 1.0–80.0 µg of TTX released from these polymers produced a range of durations of nerve block, from several hours to 3 days, with minimal systemic or local toxicity.

## Abstract (web summary)

There is interest in developing long-lasting local anaesthetics for a range of applications. Here, the authors report on the application of tetrodotoxin conjugated to amphiphilic biodegradable polymer to reduce systemic toxicity, achieve sustained release and investigate application as a local anaesthetic.

## Introduction

Opioids are often the mainstay of perioperative and chronic pain management, even with relatively localized pain. Opioids have numerous side effects, including nausea, clouding of the sensorium, pruritus, urinary retention, and constipation. More seriously, opioid therapy often results in tolerance, addiction, diversion, and fatal overdose. Opioids are often delivered systemically, and act predominantly on the central nervous system, the origin of the major side effects. Consequently, there has been growing interest in local and regional approaches to treating pain.

Conventional amino-amide and amino-ester local anesthetics are the principal compounds used for local and regional anesthesia^1,2^. Although these compounds are effective, their durations of action are relatively short, and they can cause severe cardiovascular and neurologic systemic side effects^1,3^. Encapsulation in sustained release systems can prolong the duration of nerve blockade of conventional local anesthetics, and can greatly mitigate systemic effect. However, it can exacerbate the intrinsic myo- and neurotoxicity of conventional local anesthetics, and can leave debris at the nerve, causing inflammation that can outlast the duration of nerve blockade^1,3,4^.

Given these limitations, it has been a long-standing goal to develop a local anesthetic sustained release system that can provide prolonged peripheral nerve blockade with minimal systemic and local side effects. Naturally occurring site 1 sodium channel blockers (S1SCBs), such as tetrodotoxin (TTX), have been studied for this purpose, as they are extremely potent and cause minimal local toxicity^5^. Consequently, S1SCBs are being investigated clinically for local anesthesia^6,7^. However, the dosing of S1SCBs, and therefore the maximal durations of block achievable, can be limited by systemic toxicity^8^. Mitigation of that toxicity by encapsulation is challenging due to the hydrophilicity of S1SCBs; in particular the initial burst release can be dose-limiting^9,10^. We have hypothesized that covalent conjugation of TTX onto a biodegradable and biocompatible polymer backbone via a hydrolyzable ester linkage would prevent the initial burst release of TTX, and that the slow hydrolysis of ester bonds would achieve sustained release of TTX in its native, biologically active form. Since the hydrophilicity of polymers is a primary determinant of the hydrolysis rate of ester bonds and therefore the TTX release from these polymers, the TTX release rate could be tailored by altering the hydrophilicity of the polymer backbone^11^.

**Fig. 1:**
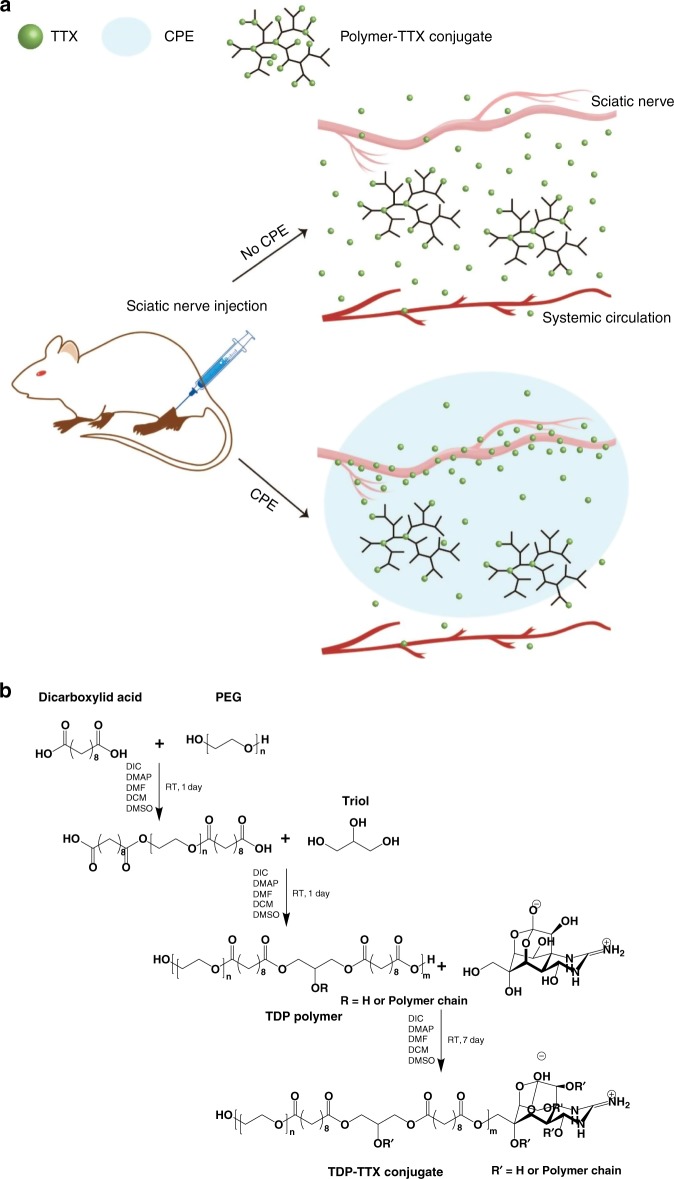
Design of a sustained release system for prolonged duration local anesthesia. **a** A polymer–TTX conjugate, designed to have a large TTX content with slow release, is placed near a nerve. Flux of TTX into the nerve is enhanced by a delivery system that acts as a chemical permeation enhancer. The polymer–TTX conjugate, schematized here as a hyperbranched structure has a much higher TTX loading than those actually produced here (which have 0.008 or 0.03 TTX molecules on each polymer chain). **b** Steglich esterification to synthesize TDP and TDP–TTX. The first step is optional, depending on whether the PEG moiety needs to be incorporated into the polymer

We hypothesized that safety and efficacy could be enhanced by minimizing the amount of S1SCB released—even to subanesthetic levels—but to additionally provide a means of enhancing the effectiveness of released drug to anesthetic levels (Fig. 1a). The approach we selected was based on the fact that only a very small fraction (perhaps 0.05%) of S1SCBs applied outside a nerve bundle penetrates to the axonal surface^4^. The penetration of S1SCBs into nerve can be enhanced by chemical permeation enhancers (CPEs)^12,13^, a heterogeneous group of (usually) small molecules that can help drugs cross biological barriers^14^.

Here, we selected TTX as the model S1SCB as it is commercially available. For the sustained release component of this system, we designed a family of biodegradable poly(triol dicarboxylic acid)-poly(ethylene glycol) (TDP) polymers, to which TTX would be covalently conjugated by ester bonds. We investigated the release of TTX in its native state from the polymers, as a function of monomer composition and polymer hydrophilicity, and studied anesthetic efficacy and toxicity in relation to those properties. The family of polymers so developed could produce nerve blocks in vivo lasting hours to days with minimal local and systemic toxicities.

## Results

### TDP polymer

TDP polymers were synthesized from dicarboxylic acids, triols, and hydroxyl poly(ethylene glycol) (PEG). The dicarboxylic acids provide carboxyl groups for TTX conjugation. The three hydroxyl groups of the triols allow the possibility of forming hyperbranched polymers. Hyperbranched TDP would terminate with multiple carboxyl groups, to which TTX could be covalently conjugated via ester bonds. TTX could be subsequently released via the hydrolysis of those bonds (Fig. 1a). The hydrophilicity of TDP polymers and hence the TTX release rate could be regulated by tuning the composition of monomers.

The TDP polymers were formed via Steglich esterification at room temperature (Fig. 1b, Supplementary Table 1). The hydrophilicity of the polymers was modified by using a hydrophilic (glycerol) or hydrophobic (polycaprolactone, PCL) triol, altering the number of carbons (1, 5, or 8) in the aliphatic chain of dicarboxylic acid, and varying the molecular weight of PEG. The nomenclature of synthesized TDP polymers will be T_*x*_D_*y*_P_*z*_, where *x* represents the type of triol (g is glycerol, c is PCL triol), *y* represents the number of carbons in the aliphatic chain of dicarboxylic acid, and *z* represents the molecular weight of PEG (200, 1000, 2000 Da) (Table 1). The presence of glycerol and PEG within TDP polymers was intended to increase the hydrophilic fraction (*f*_phil_) of the polymers, defined as the weight percentage of glycerol and PEG within the TDP polymer.

**Table 1:** Characterization of TDP polymers

Name^a^	*f*_phil_ (%)^b^	Dicarboxylic acid	Triol	PEG	Mn^c^	Mw^c^	PDI^c^
T_g_D_8_P_2000_	83.5	Sebacic acid	Glycerol	PEG_2000_	6643	11,234	1.691
T_g_D_8_P_1000_	72.1	Sebacic acid	Glycerol	PEG_1000_	5578	7672	1.375
T_g_D_8_P_200_	37.8	Sebacic acid	Glycerol	PEG_200_	4470	7081	1.584
T_g_D_1_	34.0	Malonic acid	Glycerol	–	4567	11,628	2.546
T_g_D_5_	28.9	Glutaric acid	Glycerol	–	5962	16,595	2.783
T_g_D_8_	21.0	Sebacic acid	Glycerol	–	6011	16,564	2.756
T_c_D_8_	0	Sebacic acid	PCL triol^d^	–	6126	17,073	2.787

^a^The polymer names are abbreviated or simplified as described in the main text

^b^Hydrophilic fraction of the polymers (*f*_phil_): weight percentage of PEG and glycerol within the polymer

^c^As determined by GPC

^d^PCL triol = polycaprolactone triol

**Fig. 2:**
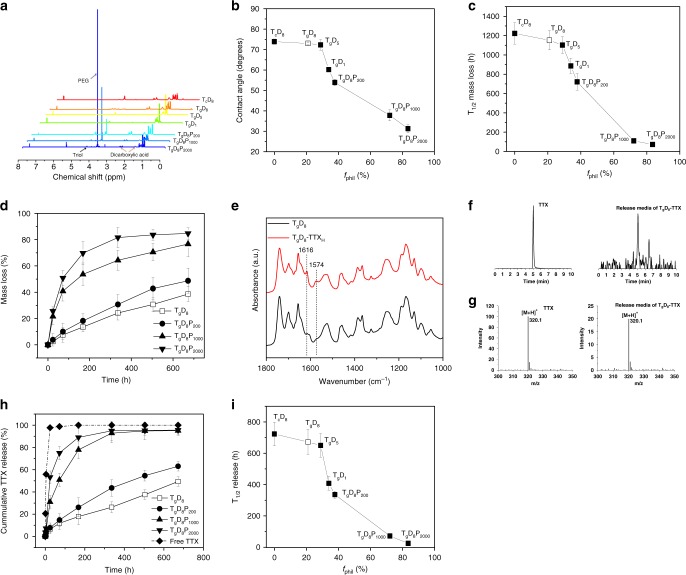
Characterization of the TD/TDP polymers and their TTX conjugates. **a** ^1^H-NMR spectra of the TD and TDP polymers. **b** Effect of polymer hydrophilicity (*f*_phil_) on contact angle of the polymer thin film. **c** Effect of *f*_phil_ on half time of mass loss. **d** Degradation profiles of the TD and TDP polymers. **e** FTIR spectra of the T_g_D_8_ polymer and T_g_D_8_–TTX_H_ conjugates. The guanidinium group peak is characteristic of TTX. **f** Total ion chromatograms of a TTX standard (10 µg mL^−1^) in citrate buffer and release media from T_g_D_8_–TTX conjugates. Both show a single peak at ~5.0 min. **g** Mass spectra confirm that the molecular weight of the peak observed at ~5.0 min corresponds to that of TTX (*m*/*z* 320.1 is [TTX + H]^+^). **h** Release profiles of 10 µg of free TTX or TTX from TD–TTX and TDP–TTX conjugates with differing *f*_phil_. **i** Release half-time of 10 µg of conjugated TTX as a function of *f*_phil_ of polymer. In panels (**a**–**d**), (**h**), and (**i**), the open box denotes T_g_D_8_. Data in graphs are means ± SD, *n* = 4

The TDP polymers were solids. In the ^1^H NMR of TDP polymers, the methylene peaks of dicarboxylic acid were detected at 1.30, 1.62, and 2.35 ppm^15^, and methylene peaks of triol were detected between 4.05 and 4.35 ppm (Fig. 2a)^15^. In T_g_D_8_P_200_, T_g_D_8_P_1000_, and T_g_D_8_P_2000_, an additional methylene peak was observed between 3.45 and 3.60 ppm, indicating a PEG segment^15^. The molecular weights of TDP polymers were determined by gel permeation chromatography (GPC) (Table 1). The molecular weights (Mn) of TDP polymers were 4000–7000 Da.

The hydrophilicity of the TDP polymers was investigated by assessing surface and bulk characteristics. The surface characteristics of polymer thin films were assessed by contact angle goniometry. An increase in the polymers’ *f*_phil_ correlated inversely with the contact angle (Fig. 2b): as *f*_phil_ increased from 0 to 83.5%, the contact angle of the polymer thin film decreased from 73.9 ± 1.3 to 31.3 ± 2.1° (means ± SD; *n* = 4). Increasing *f*_phil_ was associated with greater water solubility (Supplementary Fig. 1).

The in vitro degradation rate of polymers increased with increasing *f*_phil_ in phosphate buffered saline (PBS, pH 7.4, 37 °C) (Fig. 2c), presumably because a higher *f*_phil_ would result in higher water uptake and accelerate ester bond hydrolysis. When the *f*_phil_ was below 37.8% (T_g_D_8_ and T_g_D_8_P_200_), polymers followed a near linear mass loss (Fig. 2d).

Myotubes from the myoblast C2C12 cell line were used to assess potential myotoxicity^16^, and the pheochromocytoma PC12 cell line to assess neurotoxicity^17^. There was no decrease in cell viability in any group in either cell line over a 24-h period (Supplementary Fig. 2).

### TDP–TTX conjugates

To avoid drug degradation, Steglich esterification, which occurs at room temperature, was selected for the synthesis of TDP–TTX conjugates (Fig. 1b). DMSO was chosen as the solvent because TTX had its highest solubility in DMSO (10 µg mL^−1^) among the tested organic solvents (Supplementary Table 2). 1–4 mg (0.003–0.012 mmol) of TTX was added into the reaction mixture (see Methods, Table 2). Only a small amount of TTX was originally dissolved in DMSO and participated in the reaction. However, as the Steglich esterification reaction proceeded and dissolved TTX was conjugated to TDP polymer, the solution equilibrium drove more TTX to dissolve and participate in the reaction. To achieve a high proportion of binding of TTX to TDP, TTX was reacted with excess TDP polymers (Table 2), the reaction occurred at room temperature for 7 days, and TDP–TTX conjugates were collected as yellowish solids. The proportion of bound TTX was determined by measuring the unbound TTX in the reaction mixture by ELISA. >99.0% of the TTX was conjugated to TDP polymer (Table 2, Supplementary Table 3). Given the potency of TTX, milligram quantities of TTX were conjugated to gram quantities of TDP polymers. The TTX loading of the synthesized TDP–TTX conjugates was in the range of 0.10–1.60 µg mg^−1^ (Table 2).

**Table 2:** Characterization of TDP–TTX conjugates

Conjugate	Feed dicarboxylic acid (mmol)	Feed triol (mmol)	Feed PEG (mmol)	Feed TTX (mmol)	TTX loading^a^ (µg mg^−1^)	Proportion of bound TTX^b^ (wt%)
T_g_D_8_P_2000_–TTX	5	1.25	2.5	0.003	0.16	99.2
T_g_D_8_P_1000_–TTX	5	1.25	2.5	0.003	0.28	99.0
T_g_D_8_P_200_–TTX	10	2.5	5	0.003	0.31	99.5
T_g_D_8_–TTX	10	5.8	–	0.003	0.40	99.4
T_g_D_8_–TTX_H_^c^	10	5.8	–	0.012	1.60	99.4
T_g_D_5_–TTX	10	5.8	–	0.003	0.54	99.5
T_g_D_1_–TTX	10	5.8	–	0.003	0.64	99.4
T_c_D_8_–TTX	10	5.8	–	0.003	0.27	99.6

^a^As determined by ELISA

^b^Proportion of TTX conjugated with polymer as a fraction of the total TTX in the reaction

^c^T_g_D_8_–TTX_H_ has four-fold higher TTX loading than T_g_D_8_–TTX

The conjugation of TTX to TDP polymer was confirmed by Fourier-transform infrared spectroscopy (FTIR) (Fig. 2e). For example, after conjugation with TTX, T_g_D_8_ exhibited new peaks at 1574 and 1616 cm^−1^, which can be assigned to the guanidium group of TTX^18^.

There was no obvious increase in the molecular weight of TDP polymers after TTX conjugation (Supplementary Table 4) because TTX loading was very low: 0.40 µg TTX per mg polymer in T_g_D_8_–TTX and 1.60 µg TTX per mg polymer in T_g_D_8_–TTX_H_, corresponding to 0.008 and 0.03 TTX molecules per polymer chain for T_g_D_8_–TTX and T_g_D_8_–TTX_H_, respectively.

Release kinetics were studied in vitro in PBS (pH 7.4, 37 °C). Liquid chromatography-mass spectrometry (LC-MS) revealed a peak at ~5.0 min in release samples and the TTX standard (Fig. 2f). Mass spectra confirmed that the molecular weight of the molecule at this retention time corresponded to that of TTX (*m*/*z* 320.1 is [TTX + H]^+^) (Fig. 2g), indicating that TTX was released in its native form. All TDP–TTX conjugates significantly increased the duration of TTX release compared to free TTX (Fig. 2h, i). TTX release followed a near linear profile at lower values of *f*_phil_ (T_g_D_8_–TTX and T_g_D_8_P_200_–TTX), suggesting the potential to release TTX at a constant rate for prolonged local anesthesia with minimal systemic toxicity. As the *f*_phil_ decreased from 83.5 to 0%, the TTX release half-time increased from 25 ± 5 h to 723 ± 75 h.

To demonstrate that TTX release was dependent on the cleavage of ester bonds, TTX was conjugated to T_g_D_8_ via urethane bonds (Supplementary Figs. 3 and 4a), which are hydrolyzed at a much slower rate than ester bonds^19,20^. In vitro, after 28 days of incubation in PBS (pH 7.4, 37 °C), no ELISA-detectable TTX was released from 25 mg of T_g_D_8_–TTX urethane conjugate containing 10 μg of TTX (Supplementary Fig. 4b).

The synthetic approach used here can be extended to covalently conjugate other drugs containing hydroxyl or carboxyl groups with TDP polymers via hydrolyzable ester bonds. As an example, we conjugated dexamethasone to TD and TDP polymers (Supplementary Figs. 5 and 6, Supplementary Table 5).

### Fabrication of syringe-injectable formulation

Injectability is a crucial property for local anesthetics. T_g_D_8_P_2000_, with a high *f*_phil_ of 83.5% could form a homogeneous injectable solution in PBS, but other TDP polymers with a low *f*_phil_ could not be homogeneously suspended in PBS (Supplementary Fig. 1). In order to render TDP–TTX conjugates injectable, they were dissolved in DCM and PEG (200 Da, PEG200), followed by solvent evaporation (Supplementary Fig. 7). The resultant formulation is denoted TDP-TTX/PEG 200.

Viscosity is an important factor for syringeability and injectability. The complex viscosity, dynamic storage (G′) and loss (G″) moduli of the TDP–TTX/PEG200 formulations were characterized at a range of angular frequencies (Supplementary Fig. 8). At 50 mg mL^−1^, all TDP–TTX/PEG200 formulations had a viscosity less than 10 Pa s in the range of angular frequencies tested (Supplementary Fig. 8a). G″ was higher than G′, indicating that the viscous component of the complex modulus dominated the materials’ behaviors, i.e., they behaved as liquids (Supplementary Fig. 8b). The fluid-like behavior and low viscosity of TDP–TTX/PEG200 formulations indicated that they are syringe-injectable. The most viscous solution used in this work, T_g_D_8_–TTX/PEG200 (50 mg mL^−1^), was injectable, as shown in Supplementary Movie 1.

### Rat sciatic nerve blockade in vivo

Rats (4 in each group) were injected at the left sciatic nerve with 0.5 mL of specified carriers containing free TTX or TDP–TTX conjugates. They then underwent neurobehavioral testing to determine the duration of functional deficits (i.e., sensory and motor nerve blockade) in both hindpaws. The duration of deficits on the injected (left) side reflected the duration of nerve block. Deficits on the uninjected (right, contralateral) side reflected systemic TTX distribution.

**Fig. 3:**
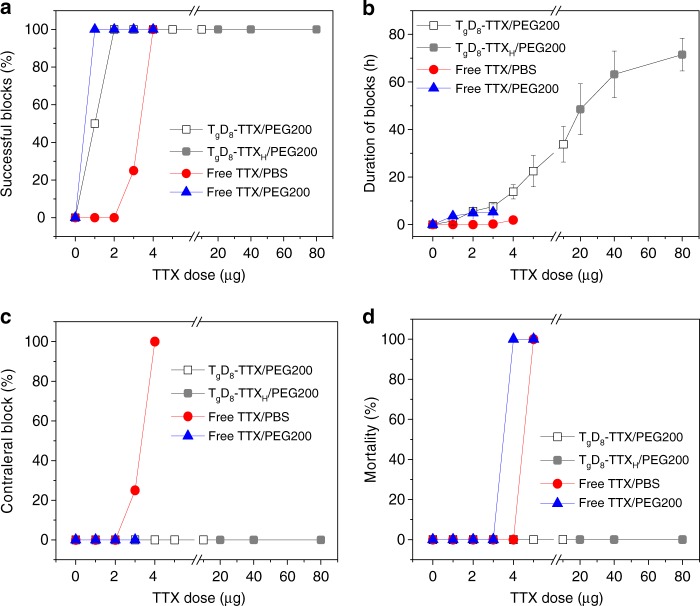
Peripheral nerve blockade with free TTX and T_g_D_8_–TTX conjugate. Effects of TTX dose on **a** frequency of successful blocks, **b** duration of sensory nerve blocks, **c** frequency of nerve blocks in the uninjected (contralateral) extremity, and **d** animal mortality. In all panels, the open box denotes T_g_D_8_–TTX, the gray box denotes T_g_D_8_–TTX_H_. Data are means ± SD, *n* = 4

Groups of rats receiving sciatic nerve injections of free TTX showed dose-dependent increases in the frequency of successful nerve blocks (Fig. 3a; see Methods for definition of success) and in the median duration of nerve block (Fig. 3b, Supplementary Fig. 9). Low doses of TTX in PBS (1 or 2 µg; 6 or 12 µM, respectively) caused no detectable nerve block or toxicity. Block from 3 µg (18 µM) of free TTX in PBS was successful in 25% of animals and produced a median duration of sensory nerve block of 0.25 ± 0.5 h; block from 4 µg (24 µM) of free TTX in PBS was successful in 100% of animals and produced a median duration of sensory nerve block of 1.9 ± 1.0 h; this is comparable to block from 0.5% bupivacaine—a commonly used anesthetic^4^. However, blocks with 4 µg of TTX in PBS caused marked systemic toxicity, as evidenced by sensory deficits in the uninjected (contralateral) leg (Fig. 3c, Supplementary Fig. 10). Injection with 5 µg (30 µM) of free TTX in PBS caused contralateral deficits in all animals and was uniformly fatal (Fig. 3d). There was no statistically significant difference between the durations of sensory and motor nerve blockade at any dose of free TTX in PBS (*p* = 1.000, one-way ANOVA) (Supplementary Fig. 9a).

Part of our design included co-delivering the polymers with a CPE. 32 mM sodium octyl sulfate (SOS) was injected with 3 µg (18 µM) of free TTX in 0.5 mL of PBS at the sciatic nerve; that concentration of SOS had been shown to prolong nerve block from TTX^12,13^. SOS increased the frequency of successful block to 100%, and increased block duration to 6.3 ± 0.9 h (*p* ≪ 0.001 compared to PBS, one-way ANOVA) (Supplementary Table 6). Since the TDP polymers were to be delivered in PEG200, we studied the effect of PEG200 on nerve block from TTX. 3 µg (18 µM) of free TTX in 0.5 mL of PEG200 resulted in 100% blockade with a duration of 5.3 ± 0.3 h (Fig. 3a, b). The improvement in success rate and duration of nerve block with PEG200 were comparable to the effects of 32 mM SOS (*p* = 0.071, one-way ANOVA). A similar prolongation of nerve block duration by PEG200 was seen with 1 and 2 µg of TTX (Fig. 3b). There was no statistically significant difference between the durations of sensory and motor nerve blockade at any dose of free TTX in PEG200 (*p* = 1.000, one-way ANOVA) (Supplementary Fig. 9a).

**Fig. 4:**
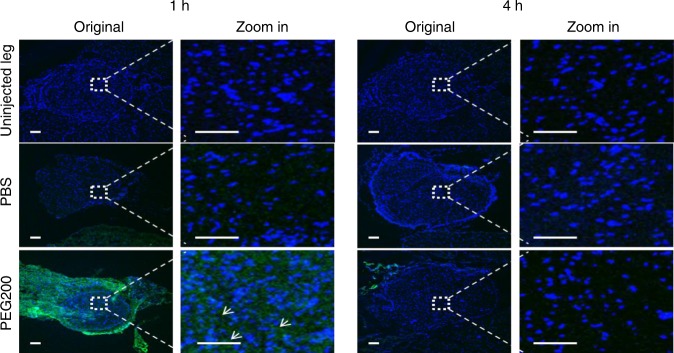
Representative fluorescence images of sections of sciatic nerves and surrounding tissues. Tissues were harvested 1 and 4 h after injection of 0.25 mg of fluorescein sodium in 0.5 mL of PEG200 or PBS. Arrows indicate fluorescein sodium (green). Blue: cell nuclei stained with 4′,6-diamidino-2- phenylindole (DAPI). Data are representative of 4 animals in each group. Scale bars, 100 µm

The fact that low doses of TTX in PEG200 had a greater rate of successful blocks than did free TTX in PBS was consistent with a CPE-like effect. (Else one would generally expect the free drug to have a higher rate of successful blocks because the initial free fraction of drug is higher.) To investigate whether PEG200 acted as a CPE, fluorescein sodium (excitation 460 nm, emission 515 nm) was used as a proxy for TTX because both are very hydrophilic. Animals were injected at the rat sciatic nerve with 0.25 mg of fluorescein sodium in 0.5 mL of PEG200 or PBS. One or four hours later, animals were euthanized and the sciatic nerve and surrounding tissue were harvested. Frozen sections of the nerve were produced, the nuclei stained, and fluorescent images taken (Fig. 4). No fluorescence was observed in the nerve in animals injected with fluorescein sodium in PBS at either time point. In animals injected with fluorescein sodium in PEG200, fluorescence was observed throughout the nerve 1 h after injection, but none 4 h after injection. These results demonstrated that PEG200 can act as a CPE, to help molecules penetrate into the nerve. Since PEG200 acted as a CPE, and it was needed to solubilize the polymers to make them injectable, it was used as the CPE in subsequent experiments.

To enhance safety and prolong release of TTX from TDP–TTX for a given loading, we selected T_g_D_8_–TTX/PEG200 for in vivo testing. Animals were injected at the left sciatic nerve with 2.5–25 mg of T_g_D_8_–TTX containing 1–10 µg of TTX, or, if higher doses were needed, with 12.5–50 mg of T_g_D_8_–TTX_H_ containing 20–80 µg of TTX (Supplementary Table 7), in 0.5 mL of PEG200. T_g_D_8_–TTX_H_ was used in order to be able to increase the TTX dose without excessive increases in the mass of polymer injected. TTX release kinetics from T_g_D_8_–TTX and T_g_D_8_–TTX_H_ were similar (*p* > 0.05, one-way ANOVA) (Supplementary Fig. 11), perhaps because the loading of TTX (1.6% of total mass) was still very low.

Efficacy, safety, and nerve block duration were greatly enhanced by covalent incorporation of TTX in T_g_D_8_/PEG200 (Fig. 3a, b). Sensory block success and duration increased with increasing doses of conjugated TTX. In particular, T_g_D_8_–TTX/PEG200 enhanced the efficacy of small doses of TTX, e.g., enabling 2 µg (12 µM) of conjugated TTX in T_g_D_8_–TTX/PEG200 to produce block with 100% success, and a median block duration of 5.6 ± 0.8 h (*p* « 0.001 compared to block duration from 2 µg of free TTX, one-way ANOVA). In addition, T_g_D_8_–TTX/PEG200 significantly increased the safety of TTX as evidenced by the absence of toxicity after injection of high doses of TTX. For example, sensory nerve block with 80 µg (480 µM) of TTX conjugated in T_g_D_8_–TTX_H_/PEG200 lasted 71.5 ± 6.9 h (approximately 3 days; Fig. 3b) and no animals died or had contralateral deficits (Fig. 3c, d). That dose of TTX was 16-fold higher than the dose of free TTX that was uniformly fatal. Moreover, in this animal model it is not possible, due to limiting toxicity, to achieve such long nerve blocks with TTX in the absence of sustained release^4^, CPEs^12,13^, and/or drugs that enhance the effect of S1SCBs^9,21^. Motor block was not statistically significantly longer than sensory block at any dose of conjugated TTX (Supplementary Fig. 9b).

The unconjugated mixture of T_g_D_8_ with free TTX in PEG200 provided similar nerve block and toxicity as did free TTX in PEG200 (Supplementary Table 6), indicating that the covalent bonding of TTX to T_g_D_8_ had a marked impact on nerve block and safety (Supplementary Note 1), and that T_g_D_8_ had no CPE-like effect (Supplementary Note 2). Separate experiments showed that nerve block depended on both the sustained release of TTX from T_g_D_8_–TTX_H_ and the CPE effect of PEG200 (Supplementary Note 3).

A crucial hypothesis underlying this work was that TTX bound to a polymer would be inactive. To test this hypothesis, 10 μg of TTX conjugated to T_g_D_8_ via urethane bonds were injected at the sciatic nerve. No sensory or motor nerve blockade was produced in any of the animals tested (Supplementary Fig. 4c). These results indicated that TTX had no biological activity when covalently conjugated onto a polymer backbone.

**Fig. 5:**
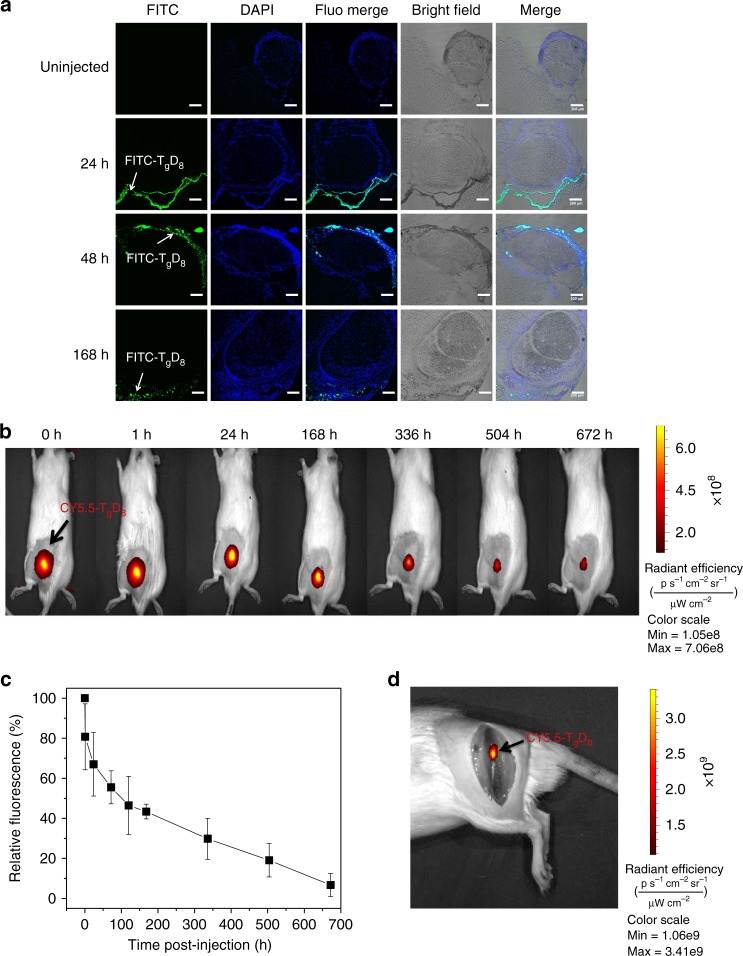
Retention in tissue of fluorescently labeled T_g_D_8_ injected at the sciatic nerve. **a** Representative fluorescent confocal photomicrographs 24, 48, and 168 h after sciatic nerve injection of 25 mg of FITC–T_g_D_8_ in 0.5 mL of PEG200. Green: FITC–T_g_D_8_; blue: cell nuclei (stained with DAPI). Scale bars, 200 µm. **b** Whole body imaging of fluorescence injection of 25 mg of CY5.5–T_g_D_8_ in 0.5 mL of PEG200. Fluorescence intensity is represented as radiant efficiency. **c** Quantification of data in panel (**b**), Data are means ± SD (*n* = 4). **d** Representative photographs of dissection of injection site 24 h after injection. Data are representative of 4 animals in each group

To address the possibility that the enhanced nerve block with T_g_D_8_–TTX/PEG200 was due to translocation of T_g_D_8_–TTX into the sciatic nerve, we labeled T_g_D_8_ with fluorescein isothiocyanate (FITC, excitation 488 nm and emission 519 nm) and injected animals with FITC-labeled T_g_D_8_ in 0.5 mL PEG200. The FITC was covalently conjugated (see Methods) to T_g_D_8_ so that the dye would not be able to diffuse independently of the polymer: the labeled polymer is denoted FITC–T_g_D_8_. At predetermined time points after injection, animals were euthanized and the nerves and surrounding tissues processed for histology. Fluorescent confocal microscopy showed FITC in the connective tissue between muscle and nerve 24, 48, and 168 h after injection, but not within the sciatic nerve itself (Fig. 5a).

To evaluate the time course of local retention of TDP in tissue, we injected animals with T_g_D_8_/PEG200 to which Cy5.5 (excitation 684 nm, emission 710 nm) was covalently conjugated; the polymer is denoted Cy5.5–T_g_D_8_. Fluorescent images were taken by an in vivo imaging system (IVIS). A fluorescent signal was seen at the sciatic nerve of all animals, with no detectable fluorescence elsewhere (Fig. 5b). The fluorescence signal decreased over 4 weeks, indicating the gradual degradation of T_g_D_8_ (Fig. 5b, c). On necropsy 24 h after injection, visible deposits were located at the sciatic nerve; these were fluorescent, confirming that they contained T_g_D_8_ (Fig. 5d).

**Fig. 6:**
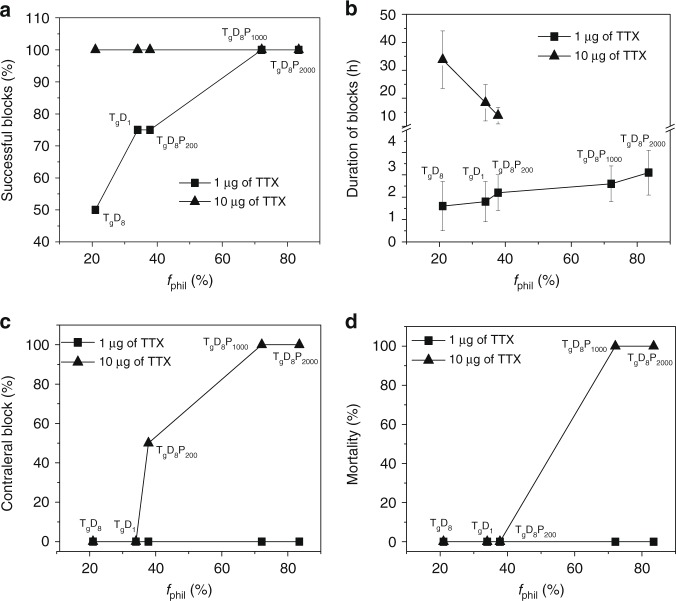
Effects of *f*_phil_ (%) on efficacy and toxicity of sciatic nerve block. **a** Frequency of successful blocks, **b** duration of sensory nerve blocks, **c** frequency of nerve blocks in the uninjected (contralateral) extremity, and **d** animal mortality. Animals were injected with 1 or 10 µg of conjugated TTX in TD–TTX/PEG200 or TDP–TTX/PEG200. Data are means ± SD, *n* = 4

Since the *f*_phil_ of TDP polymers determined the TTX release rate from TDP–TTX conjugates in vitro, we hypothesized that it would determine the toxicity and efficacy of the formulations in vivo. To test our hypothesis, animals were injected at the left sciatic nerve with TD–TTX/PEG200 or TDP–TTX/PEG200 conjugates with various *f*_phil_ containing 1 (6 µM) or 10 µg (60 µM) of TTX (Supplementary Table 7). In animals injected with 1 µg of conjugated TTX, the frequency of successful blocks and the block duration increased with increasing *f*_phil_ (Fig. 6a, b). In animals injected with 10 µg of conjugated TTX, the toxicity (sensory deficits in the uninjected [contralateral] leg and mortality) increased, while the block duration decreased with increasing *f*_phil_ (Fig. 6c, d). These results demonstrated that the *f*_phil_ of TDP polymers determined the efficacy and toxicity of the formulations. At a low dose of TTX (1 µg conjugated to TDP polymers), a relatively rapid release of TTX was required to achieve and maintain the tissue level of anesthetic required to provide nerve block. This was more readily achieved with molecules with a high *f*_phil_, which have more rapid drug release. However, at a high dose of conjugated TTX (10 µg), where the concentration of released TTX readily achieved that needed for effective nerve block, more rapid release of TTX could cause systemic toxicity, and a shorter duration of effect. Therefore, at higher TTX doses a lower *f*_phil_ would enable a slower rate of TTX release to minimize systemic toxicity and prolong block duration.

Since inadvertent intravascular injection can cause systemic anesthetic toxicity, the safety of intravenous polymer–TTX/PEG200 was assessed. T_g_D_8_-TTX_H_/PEG200 solution (50 mg mL^−1^ T_g_D_8_–TTX_H_) was diluted 1:1 in PBS containing 10 wt% BSA, to a final concentration of 25 mg mL^−1^ T_g_D_8_–TTX_H_. 0.5 mL of the formulation (12.5 mg of T_g_D_8_–TTX_H_) containing 20 µg (120 µM) of TTX was injected intravenously. There were no neurobehavioral deficits at the sciatic nerve (which would reflect systemic toxicity) or other signs of toxicity, including animal death (Supplementary Table 9). Notably, 20 µg of intravenous TTX is enough to kill many rats, since the LD_50_ of intravenous TTX is 2–10 µg kg^−1^ in mammals^22^, i.e., 0.7–3.5 µg in a 350 g rat.

### Tissue reaction

**Fig. 7:**
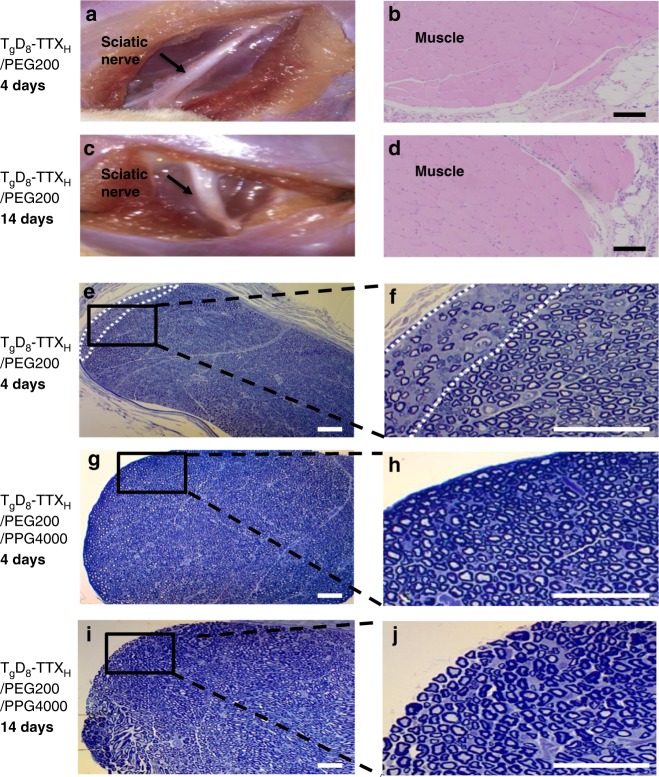
Tissue reaction to materials. **a**, **c** Representative photographs of the site of injection upon dissection 4 and 14 days after injection of 25 mg of T_g_D_8_–TTX_H_ in 0.5 mL of PEG200. **b**, **d** Representative hematoxylin–eosin stained sections of muscles and adjacent loose connective tissue 4 and 14 days after injection of 25 mg of T_g_D_8_–TTX_H_ in 0.5 mL of PEG200. Scale bars, 100 µm. **e**–**j** Toluidine blue stained sections of nerve after injection of formulated T_g_D_8_–TTX_H_. Scale bars, 50 µm. **e**, **f** Minimal peripheral injury (area enclosed by white dotted lines) seen in 1 of 3 nerves 4 days after injection of 25 mg of T_g_D_8_–TTX_H_ in 0.5 mL of PEG200. **g**–**j** Representative toluidine blue stained sections of nerve 4 days (**g**, **h**) and 14 days (**i**, **j**) after injection of 25 mg of T_g_D_8_–TTX_H_ in 0.5 mL mixture of PEG200 and PPG4000 (5/95, v/v), showing no injury. Data are representative of 3 animals in each group

Animals injected with free TTX and TDP–TTX conjugates with various *f*_phil_ (Table 3) were euthanized 4 and 14 days after sciatic nerve injection. Representative tissue reaction to T_g_D_8_–TTX_H_ conjugates is shown in Fig. 7. At dissection, there was no obvious residual material although it was seen on confocal microscopy and IVIS (Fig. 5). The tissues did not appear edematous or discolored, and had no other gross signs of tissue injury (Fig. 7a, c). Sciatic nerves and surrounding tissues were sectioned and harvested for histologic evaluation. Muscle tissue was processed for hematoxylin–eosin (H&E) staining. CPEs, including SOS, can cause concentration-dependent myotoxicity and inflammation^10,11^. Microscopic examination did not reveal significant myotoxicity or inflammation in animals in any group at 4 or 14 days after injection (relevant scores^12^ in Table 3, images in Fig. 7b, d). There was no statistically significant difference between the scores in any group injected with TDP–TTX and TTX alone.

**Table 3:** Myotoxicity and inflammation

Formulation	Dose of polymer (mg)	Dose of TTX (µg)	Myotoxicity score (range) Day 4	Myotoxicity score (range) Day 14	Inflammation score (range) Day 4	Inflammation score (range) Day 14
Free TTX^a^	–	3.0	0 (0–1.0)	0 (0–1.0)	0 (0–1.0)	0 (0–1.0)
PEG200	562^b^	3.0	0 (0–1.0)	0 (0–1.0)	0 (0–1.0)	1.0 (0–1.0)
*p* Value			1.000	1.000	1.000	0.456
T_g_D_8_–TTX_H_	50^c^	80.0	1.0 (0–1.0)	1.0 (0–1.0)	1.0 (0–1.0)	1.0 (0–1.0)
*p* Value			0.456	0.456	0.456	0.456
T_g_D_1_–TTX	12.5^c^	31.9	1.0 (0–1.0)	1.0 (0–1.0)	0 (0–1.0)	1.0 (0–1.0)
*p* Value			0.456	1.000	1.000	0.456
T_g_D_8_P_200_–TTX	32.5^c^	10.0	0 (0–1.0)	0 (0–1.0)	1.0 (0–1.0)	0 (0–1.0)
*p* Value			1.000	0.456	0.456	1.000
T_g_D_8_P_1000_–TTX	25^c^	6.9	0 (0-0)	0.5 (0–1.0)	0.5 (0–1.0)	0.5 (0–1.0)
*p* Value			0.414	0.738	0.738	0.738
T_g_D_8_P_2000_–TTX	25^c^	3.5	0.5 (0–1.0)	0 (0–0)	0.5 (0–1.0)	0.5 (0–1.0)
*p* Value			0.738	0.182	0.738	0.738

Data are medians with interquartile ranges. *P* values are for the comparison of the tissue reaction of test compounds to that of free TTX. *n* = 3 per experimental group. Inflammation scores range: 0–4; myotoxicity scores range: 0–6

^a^3 µg of free TTX in 0.5 mL of PBS

^b^3 µg of free TTX in 0.5 mL of PEG200

^c^TD–TTX and TDP–TTX conjugates were formulated in 0.5 mL of PEG200

Since H&E-stained are relatively insensitive for identifying nerve injury, we obtained toluidine blue-stained Epon-embedded sections of the sciatic nerve in animals injected with TDP–TTX. Minimal peripheral injury (decreased density of axons) involving <5% of the nerve, was detected at 4 days in 1 of 3 rats injected with T_g_D_8_–TTX_H_/PEG200 (Fig. 7e, f).

That minimal nerve injury was due to the high concentration of PEG200 (5.62 M, i.e., pure) used to disperse the T_g_D_8_–TTX_H_, as evidenced by comparable minimal nerve injury present at 4 days in 3 of 3 rats injected with 0.5 mL of pure PEG200 (Supplementary Fig. 12a, b). We eliminated the minimal nerve injury by reformulating the injection solution. We selected compounds for the following properties: (a) ability to disperse the TTX-polymer conjugate, (b) activity as a CPE, (c) liquidity at room temperature, (d) lack of toxicity. Poly(propylene glycol) (4000 Da, PPG4000) was found to cause no nerve damage, which is consistent with reports on the safety of high molecular weight poly(propylene glycol) (2000 Da or greater)^23^. Nerve histology was normal in all rats injected with 0.5 mL of pure PPG4000 at 4 days (Supplementary Fig. 12c). However, injection of T_g_D_8_–TTX_H_ in PPG4000 containing up to 80 µg (480 µM) of TTX did not result in nerve block in rats, due to the poor CPE effect of PPG4000: confocal imaging showed that PPG4000 did not facilitate the penetration of fluorescein sodium into sciatic nerves (Supplementary Fig. 13). Consequently, we combined PPG4000 (95 v%) acting as an inert carrier with 5 v% PEG200 (281 mM) as the active CPE. Injection of T_g_D_8_–TTX_H_ containing 40 µg of TTX in 5% PEG200/95% PPG4000 induced nerve block lasting 64.8 ± 16.6 h, similar to that resulting from the T_g_D_8_–TTX_H_/PEG200 formulation (*p* = 0.88, one-way ANOVA). Nerve histology confirmed that this formulation did not cause any nerve injury at 4 and 14 days (Fig. 7g–j).

## Discussion

We had conjectured that prolonged duration local anesthesia could be achieved without significant systemic or local toxicity by combining a sustained release system eluting drug at a subtherapeutic level, with a CPE to enhance the effect of the released anesthetic. For sustained release, we developed biodegradable polymer–TTX conjugates; for the CPE we used a mixture containing 5 v% of PEG200 and 95 v% of PPG4000. This combination of materials with distinct properties was able to provide prolonged local anesthesia with no systemic toxicity.

Animal testing confirmed that TDP–TTX/PEG200 could significantly broaden the therapeutic index of TTX, i.e., reducing toxicity while increasing efficacy. All TDP–TTX/PEG200 formulations significantly increased the safety of TTX (Fig. 3, Supplementary Fig. 10). In particular, T_g_D_8_-TTX_H_/PEG200 allowed the injection of up to 80 µg of TTX without systemic toxicity; TDP–TTX/PEG200 was able to increase the efficacy of TTX, i.e. increasing the duration of nerve block that could be achieved and enhancing the effectiveness of small doses of TTX (e.g. enabling 1 µg of TTX to produce nerve blockade).

Hydrolyzable covalent bonds have been used to create drug conjugates for controlled drug release due to their excellent control over release rates and avoidance of burst release^19,20^. However, for a specific drug, precise control of the hydrolysis rate of the covalent bonds is required to maintain drugs level within a therapeutic window. TTX has a very narrow therapeutic window^24^ (Fig. 3a), which requires control of the TTX release rate to avoid systemic toxicity while prolonging block. To achieve this goal, TDP polymers with adjustable backbone hydrophilicity and therefore a tunable hydrolysis rate of ester bond were developed. Synthesis of a library of TDP–TTX conjugates with various TTX release rates, allowed the identification of TDP–TTX conjugates with desired characteristics.

Given their adjustable hydrophilicity, and their multiple hydroxyl and carboxyl ending groups, the TDP polymers have the potential to be a platform for reversible binding of a wide range of drugs with hydroxyl or carboxyl groups (such as dexamethasone, above). For highly potent drugs, such as TTX, TDP polymers with a lower *f*_phil_ may be preferred to achieve slow drug release. For drugs of lower potency, TDP polymers with a higher *f*_phil_ may be required to achieve a faster drug release rate.

TDP–TTX conjugates were biodegradable and tissue reaction was benign. All monomers used for the synthesis of TDP–TTX conjugates, including glycerol^25^, polycaprolactone^26^, dicarboxylic acid^25^, and PEG^27^ have excellent biocompatibility. TDP–TTX conjugates should gradually degrade into intermediates, e.g., TTX in its native form or linked to a small segment of TDP chains, which would eventually hydrolyze off.

PEG200, a solvent with low toxicity, has been widely used in a variety of pharmaceutical formulations as a medium for hydrophobic pharmaceuticals^19^. We discovered that PEG200 was both a vehicle and a CPE. PEG200 enhanced TTX flux into nerve and enhanced the effectiveness of TTX, probably due to its amphiphilic nature^28,29^. Pure PEG200 caused minimal nerve injury. However, the minimal nerve injury was avoided completely by using a low yet effective concentration of PEG200 combined with PPG4000.

The TDP–TTX conjugate would be easy to scale up, weigh, and store for the future clinical applications. Milligram quantities of TTX were conjugated to gram quantities of T_g_D_8_–TTX_H_ (4 mg of TTX in 2500 mg of T_g_D_8_–TTX_H,_ Table 2). Such a batch would allow the delivery of 100 doses of 25 mg of T_g_D_8_-TTX_H_ containing 40 µg of TTX, which produced a duration of nerve block up to 63.2 ± 9.8 h in a rat (Fig. 3b). The reaction could be further scaled up without decreasing the proportion of binding of TTX to T_g_D_8_ polymer due to excess of T_g_D_8_ in the reaction mixture. The synthesized T_g_D_8_-TTX conjugate is solid, which could be easily stored at room temperature in a dry environment.

The formulations developed here would likely be even safer and provide longer blocks in larger animals such as humans. The rationale behind that hypothesis is that toxicity of a systemically distributed drug (TTX in this case) tracks fairly well with animal mass, while local anesthetic effect has a much weaker relationship to body size^4^. Therefore, while a sciatic nerve block in a 70 kg human may require more active material than in a 350 g rat, that increment will likely be much smaller than the 200-fold decrease in systemic toxicity in the human. In addition to this potential increase in the therapeutic index, it is likely that it will be possible to deliver much more material in a larger animal. Thus, for example, rat sciatic nerve block with bupivacaine-dexamethasone polymeric microspheres lasted a few days^30^, and lasted 10 days in sheep intercostal blocks^31^.

The T_g_D_8_–TTX/PEG200 formulation enables great dose flexibility. The TTX loading of T_g_D_8_-TTX_H_ (1.60 µg mg^−1^, 0.03 TTX molecules on each polymer chain) is still very low. If T_g_D_8_-TTX conjugates with higher loadings of TTX were required, that could be readily achieved by simply feeding larger amounts of TTX into the reaction, since the polymer component is present in excess. It should also be possible to substitute other S1SCB—such as the saxitoxins^24^—for TTX, since those also have hydroxyl groups available for reaction to the polymers. Nerve block durations even longer than those reported in this work could be achieved using approaches such as enhancing local retention (Supplementary Discussion 1) and co-administration/co-delivery with other drugs (Supplementary Discussion 2).

In conclusion, a local anesthetic system was developed that provided hours to days of nerve block with minimal local or systemic toxicity. The design entailed two key components: a rationally designed TDP–TTX conjugate that provided precise control of TTX release at a safe rate, and a CPE increasing TTX flux into nerve.

## Methods

### Reagents

Sebacic acid (99.0%), malonic acid (99.0%), glutaric acid (99.0%), polycaprolactone triol (PCL triol, 300 Da), glycerol (99.0%), PEG (200, 1000, 2000 Da), N,N′-diisopropylcarbodiimide (DIC, 99.0%), 4-dimethylaminopyridine (DMAP, 99.0%), anhydrous N,N-dimethylformamide (DMF, 99.8%), anhydrous dimethyl sulfoxide (DMSO, 99.9%), anhydrous dichloromethane (DCM, 99.8%), deuterochloroform (99.96 atom% D), sodium octyl sulfate (SOS, 95.0%), dexamethasone (98.0%), fluorescein isothiocyanate isomer I (FITC, 90.0%), fluorescein sodium salt, hexamethylene diisocyanate (HMDI, 99.0%), dibutyltin dilaurate (Sn(II), 95.0%), and PBS (pH 7.4) were purchased from Sigma-Aldrich (St. Louis, MO, USA). Cyanine5.5 carboxylic acid (Cy5.5, 95.0%) was purchased from Lumiprobe Corporation (Hallandale Beach, FL, USA). Tetrodotoxin (TTX) was obtained from Abcam (Cambridge, MA, USA). TTX ELISA kits were purchased from Reagen LLC (Moorestown, NJ, USA). Dulbecco’s minimum essential medium (DMEM), fetal bovine serum (FBS), and Penicillin Streptomycin was purchased from Thermo Fisher Scientific (Waltham, MA, USA).

### Synthesis of TDP and TD polymers

The TDP polymers were synthesized via Steglich esterification, using DIC as a coupling reagent and DMAP as a catalyst. In brief, dry PEG and sebacic acid (for feed amounts see Table 2 and Supplementary Table 1) were added to a dry round bottom flask. After adding 8 mL of anhydrous DMF, 8 mL of anhydrous DMSO and 4 mL of anhydrous DCM, and sonicating the mixture for several minutes, DIC (4.336 mL, 28 mmol) and DMAP (0.489 g, 4 mmol) were added. The mixture was kept at room temperature under nitrogen for 24 h. Glycerol (for feed amounts see Table 2 and Supplementary Table 1) was added and the mixture was left at room temperature for 24 h. After reaction, DCM in the reaction mixture was removed by rotary evaporation (Buchi R-210, Marshall Scientific, Hampton, NH, USA). The residue was precipitated with 30 mL of DI water and further washed 2 times with 30 mL of DI water containing 10 v% ethanol. The solid residue was dried by lyophilization (Virtis sentry 2.0, SP Scientific, Gardiner, NY, USA) overnight. Subsequently, the dried polymer was redissolved in DCM and purified by precipitation with 30 mL of diethyl ether. Then the supernatant was removed and the participate was dried under vacuum overnight. TDP and TD polymers, slightly yellowish solid, were obtained with yields of 88–96%. The dried TDP and TD polymers were stored in a desiccator for further use.

### Synthesis of TDP–drug and TD–drug conjugates

The TDP–drug conjugates were synthesized by Steglich esterification. In brief, dry PEG and sebacic acid (for feed amounts see Table 2 and Supplementary Table 1) were added to a round bottom flask. After adding 8 mL of anhydrous DMF, 8 mL of anhydrous DMSO, and 4 mL of anhydrous DCM, and sonicating the mixture for several minutes, DIC (4.336 mL, 28 mmol) and DMAP (0.489 g, 4 mmol) were added. The mixture was kept at room temperature under nitrogen for 24 h. Glycerol (for feed amounts see Table 2 and Supplementary Table 1) was added and the mixture was left at room temperature for 24 h. An anhydrous DMSO (8 mL) suspension of TTX (for feed amounts see Table 2 and Supplementary Table 1), and/or dexamethasone (for feed amounts see Table 2 and Supplementary Table 5), and/or FITC (1 mg, 0.003 mmol), and/or Cy5.5 (1.6 mg, 0.003 mmol) was added and the mixture was left at room temperature for 7 days. After reaction, DCM in the reaction mixture was removed by rotary evaporation. The residue was precipitated with 30 mL of DI water and further washed 2 times with 30 mL of DI water containing 10 v% ethanol. The solid residue was dried by lyophilization overnight. Subsequently, the dried polymer was redissolved in DCM and purified by precipitation with 30 mL of diethyl ether. Then the supernatant was removed and the participate was dried under vacuum overnight. TDP–drug conjugates, slightly yellowish solid, were obtained with yields of 88–96%. The dried TDP–drug conjugates were stored in a desiccator for further use.

### Synthesis of T_g_D_8_–isocyanate

In a typical synthesis, 2.4 g of T_g_D_8_ (Mn = 6011, 0.4 mmol) was dried in a 100 mL flask under vacuum overnight. Then, 5 mL of anhydrous DMSO was added to the flask, and 1.13 g of HMDI (240 µL, 1.5 mmol) and 8 mg of dibutyltin dilaurate were added sequentially. The reaction mixture was stirred at 60 °C under a nitrogen atmosphere overnight. At the end of the reaction, the resultant polymers were precipitated with diethyl ether and further purified by redissolving in DCM followed by precipitation in a mixture of methanol and diethyl ether (5/95, v/v) to remove remaining dibutyltin dilaurate^32^. Upon drying under vacuum, T_g_D_8_–isocyanate was obtained with yields 80–95%.

### Synthesis of T_g_D_8_–TTX urethane

Typically, 0.325 g of T_g_D_8_**-**isocyanate was dried in a 100 mL flask under high vacuum overnight. Then, 5 mL of anhydrous DMSO was added to the flask, and 4 mg of dibutyltin dilaurate and 0.1 mg of TTX suspended in 0.05 mL of anhydrous DMSO were added sequentially. The reaction mixture was stirred at 60 °C under a nitrogen atmosphere overnight. At the end of the reaction, the resultant polymers were precipitated with diethyl ether and further purified by redissolving in DCM followed by precipitation in a mixture of methanol and diethyl ether (5/95, v/v) to remove remaining dibutyltin dilaurate^32^. Upon drying under vacuum, T_g_D_8_–TTX urethane was obtained in 90–95% yield.

### Contact angle measurement

Water contact angle measurements were conducted for the polymer film spincoated on silicon wafer substrates with a goniometer equipped with an automatic dispenser (model 500, Rame-Hart, Succasunna, NJ, USA). The static sessile drop method was applied. A water volume of 2 μL was deposited on the sample surface and the contact angle was determined based on the images.

### ^1^H NMR measurements

Polymer and polymer–drug conjugates were analyzed using Nuclear magnetic resonance (^1^H NMR) spectroscopy (Varian 400 MHz equipped with 5 mm AutoX OneProbe and Varian 7600 autosampler) (Varian, Palo Alto, CA, USA). Polymers were dissolved in deuterochloroform and the spectra were recorded at 400 MHz. The chemical shifts (δ, in ppm) for the peaks corresponding to the hydrogens in italics in the following list of polymers are provided^15^. s/d/m indicate the shape of a peak (i.e., singlet, doublet, triplet). ^1^H NMR (T_g_D_8_) (400 MHz, CDCl3) δ/ppm: 1.30 (2 H, m, -C*H*_2_-), 1.62 (2 H, d, -C*H*_2_CH_2_O(CO)-), 2.35 (2 H, m, -C*H*_2_O(CO)-), 3.50–3.85 (2 H, m, OHC*H*_2_CHO-), 3.94 (1 H, m, -OCH_2_C*H*OH), 4.05λ–4.35 (2 H, m, -OC*H*_2_CHO-), 5.09 (1 H, s, OHCH2C*H*O-), 5.26 (1 H, s, -OCH_2_C*H*O-). ^1^H NMR (T_g_D_8_P_1000_) (400 MHz, CDCl_3_) δ/ppm: 1.30 (2 H, m, -C*H*_2_-), 1.62 (2 H, d, -C*H*_2_CH_2_O(CO)-), 2.35 (2 H, m, -C*H*_2_O(CO)-), 3.64 (2 H, m, -OC*H*_2_-), 3.94 (1 H, m, -OCH_2_C*H*OH), 4.05–4.35 (2 H, m, -OC*H*_2_CHO-), 5.09 (1 H, s, OHCH_2_C*H*O-), 5.26 (1 H, s, -OCH_2_C*H*O-). The ^1^H NMR spectrum of all TDP polymers is shown in Fig. 2a with key structural elements assigned. Some peaks cannot be assigned due to signal overlapping.

### FTIR measurements

Fourier transform infrared (FTIR) spectra were obtained using a Thermo Nicolet Nexus 870 spectrometer operated in attenuated total reflection (ATR) mode with a deuterated triglycine sulfate KBr detector. Baseline-corrected spectra were collected over 400–4000 cm^–1^ at 4 cm^–1^ resolution and averaged over 256 scans to improve the signal-to-noise ratio. Spectra were processed using the OMNIC software package (Thermo Fisher Scientific, Waltham, MA, USA).

### Molecular weight measurement

Molecular weights were determined by GPC using tetrahydrofuran as the solvent and polystyrene as standards. GPC was performed using a Waters system equipped with a 2400 differential refractometer. 515 pump, and 717-plus autosampler (Waters Corporation, Milford, MA, USA). The flow rate was 1.0 mL min^−1^.

### In vitro degradation of polymers

Mass loss studies were performed by placing polymers into a Slide-A-Lyzer MINI dialysis device (Thermo Fisher Scientific, Waltham, MA, USA) with a 10,000 MW cut-off, dialyzed against 14 mL of PBS and incubated at 37 °C on a platform shaker at 60 rpm (New Brunswick Innova 40, Eppendorf, Hamburg, Germany). At each time point, the dialysis solution was exchanged with fresh, pre-warmed PBS. 14 mL of the dialysis solution was frozen, lyophilized, and the residue mass was weighted for mass loss analysis. The mass loss half-time was calculated based on the time taken for the lost mass accumulation equals to half of the original mass loaded.

### Cell culture

Cell culture of C2C12 mouse myoblasts (American Type Culture Collection (ATCC), Manassas, VA, USA) and PC12 rat adrenal gland pheochromocytoma cells (ATCC, Manassas, VA, USA) were performed as reported^21^. In brief, C2C12 cells were cultured in DMEM with 20% FBS and 1% Penicillin Streptomycin. Cells were seeded onto a 24-well plate at 50,000 cells mL^−1^ and incubated for 10–14 days in DMEM with 2% horse serum and 1% Penicillin Streptomycin to differentiate into myotubules. PC12 cells were grown in DMEM with 12.5% horse serum, 2.5% FBS and 1% Penicillin Streptomycin. Cells were seeded onto a 24-well-plate, and 50 ng mL^−1^ nerve growth factor was added 24 h after seeding.

### Cell viability

C2C12 mouse myoblasts and PC12 rat adrenal gland pheochromocytoma cells (1 × 10^4^ per well) were incubated with various concentrations of polymer for 24 h. After incubation, cells were washed up to 5 times with PBS to remove any remaining polymers. Cell viability was determined by MTT. In brief, culture supernatants from control and polymer-containing wells were collected and cells incubated with MTT (0.5 mg mL^−1^; 3 h). The formazan was dissolved in 200 µL of DMSO and optical density measured at 550 nm. The absorbance of control wells was assumed 100% and cell viability of treated wells was determined with respect to control wells.

### Determination of equilibrium TTX solubility

Solubility studies of TTX were determined by equilibrating excess TTX in organic solvents. Assays were performed in 2 mL tubes. In each tube 1 mL of organic solvent and 1 mg of TTX were added. The TTX amount was sufficient to saturate each solvent, as evidenced by precipitation of undissolved TTX. An incubator shaker was used to keep samples at 25 °C, with agitation at 150 rpm for 72 h (until the samples achieved equilibrium^33^). Samples were then filtered through a 0.45 μm pore-size disposable capsule filter.

To determine TTX solubility in DMSO and DMF, the filtrates were diluted in PBS to a final concentration of 10% DMSO or DMF. The TTX concentration in the mixture was measured with a TTX ELISA kit, and the standard curve of ELISA was generated by dissolving free TTX in a PBS solution containing 10% DMSO or DMF.

To determine TTX solubility in DCM, 1 mL of the filtrates was transferred into a round-bottom flask and DCM was removed via rotary evaporation. 0.1 mL of citric buffer was added into the round-bottom flask to dissolve any TTX. 50 mL of the solution was diluted with 450 mL of PBS. The TTX concentration in the mixture was measured with a TTX ELISA kit.

### Determination of the proportion of drug bound to polymer

After the esterification reaction was complete, DCM in the reaction mixture was removed by rotary evaporation. Then the reaction mixture was washed with 30 mL of DI water. Polymer–drug conjugates were centrifuged at 48,384×*g* for 5 min, the supernatant was collected as supernatant #1. The polymer–drug conjugates were washed with 40 mL of DI water and centrifuged again. The supernatant was collected as supernatant #2. The TTX concentration of the collected supernatants was measured by ELISA. Dexamethasone concentration of the collected supernatants was determined by HPLC.

Proportion of drug bound to polymer was calculated as follows:

1 $${\mathrm{Proportion}}\,{\mathrm{of}}\,{\mathrm{drug}}\,{\mathrm{bound}}\,{\mathrm{to}}\,{\mathrm{polymer}} = \frac{{{\mathrm{Drug}}_{{\mathrm{feed}}} - {\mathrm{Drug}}_{{\mathrm{unbound}}}}}{{{\mathrm{Drug}}_{{\mathrm{feed}}}}} \times 100{\mathrm{\% }}$$

### Determination of drug loading

Due to high proportion of drug bound to polymer (>99.0%), drug loading was determined by the amount of drug divided by the total reactant weight fed into the esterification reaction.

2 $${\mathrm{Drug}}\,{\mathrm{loading}} = \frac{{{\mathrm{Mass}}_{{\mathrm{drug}}}}}{{{\mathrm{Mass}}_{{\mathrm{dicarboxylic}}\,{\mathrm{acid}}} + {\mathrm{Mass}}_{{\mathrm{triol}}} + {\mathrm{Mass}}_{{\mathrm{PEG}}}}} \times 100{\mathrm{\% }}$$

Drug loading per TDP molecule was calculated as follows:

3 $$\frac{{{\mathrm{Drug}}}}{{{\mathrm{TDP}}\,{\mathrm{molecule}}}} = {\mathrm{Drug}}\,{\mathrm{loading}} \, \times \frac{{{\mathrm{Mn}}_{{\mathrm{TDP}}}}}{{{\mathrm{MW}}_{{\mathrm{drug}}}}}$$

### In vitro drug release

Drug release were performed by placing polymer–drug conjugates into a Slide-A-Lyzer MINI dialysis device (Thermo Fisher Scientific, Waltham, MA) with a 10,000 MW cut-off, further dialyzed with 14 mL PBS and incubated at 37 °C on a platform shaker (New Brunswick Innova 40, 60 rpm). At each time point, the dialysis solution was exchanged with fresh, pre-warmed PBS. 0.5 mL of the dialysis solution was saved for drug analysis. The concentration of TTX in release media was quantified by ELISA. The concentration of dexamethasone was determined by HPLC (Agilent 1260 Infinity, Agilent, Santa Clara, CA, USA) using a C18 column (Poroshell 120 EC-C18, 4.6 × 100 mm, i.d. 2.7 μm, Agilent, Santa Clara, CA, USA) with a acetonitrile/water (70:30) mobile phase and a flow rate of 0.5 mL min^−1^. Dexamethasone was detected by UV absorbance at *λ* = 254 nm. The drug release half-time was calculated based on the time taken for the released drug accumulation equals to half of the drug loaded.

### LC-MS instrumentation and conditions

Analysis was performed on an Agilent 6130 Single Quadrupole LC/MS instrument. Chromatographic separation was achieved using a Kinetex Hilic column (100 × 2.1 mm, 100 Å, 2.6-μm particles; Phenomenex, Torrance, CA, USA). 0.1% (v/v) formic acid in water was used as mobile phase A and 0.1% (v/v) formic acid in acetonitrile was used as mobile phase B. The mobile phase flow rate was 500 μL min^−1^. The injection volume was 5 μL. The gradient elution method was: 90% B to 10% B from 0 to 10 min, held at 10% B from 10 to 13 min, 10% B to 90% B from 13 to 14 min.

### Fabrication of injectable formulations

To prepare a syringe-injectable TDP–TTX/PEG200 or TDP–TTX/PEG200/PPG4000 formulation, 100 mg of TDP–TTX conjugates were fully dissolved in an excess of DCM, followed by addition of a predetermined amount of PEG200 or PEG200/PPG4000 (for concentrations see Supplementary Table 7). The resulting mixture was vortexed for 1 min to obtain a uniform solution. DCM was removed via rotary evaporation, followed by vacuum at room temperature for 2 days.

### Rheological testing

The rheological properties of the TDP–TTX/PEG200 formulations were monitored using an AR2000 rheometer (TA instruments, New Castle, DE, USA) equipped with a temperature controller. A parallel plate with 20 mm diameter was used for all tests. The gap distance between the plates was 0.3 mm. Frequency sweeps ranging from 0.1 to 100 rad/s were conducted at room temperature. A constant 0.1 Pa stress was used.

### Animal studies

Animal studies were conducted following protocols approved by the Boston Children’s Hospital Animal Care and Use Committee in accordance with the guidelines of the International Association for the Study of Pain. Adult male Sprague–Dawley rats (Charles River Laboratories, Wilmington, MA, USA) weighing 350–400 g were housed in groups under a 12-h/12-h light/dark cycle with lights on at 6:00 AM.

Sciatic nerve injections were performed with a 23 G needle at the left sciatic nerve under brief isoflurane-oxygen anesthesia^4,34^. The needle was introduced postero-medial to the greater trochanter, pointing in the anteromedial direction, and upon contact with bone the formulations were injected onto the sciatic nerve. Intravenous injections were performed with a 23 G needle via the tail vein under brief isoflurane-oxygen anesthesia.

Neurobehavioural testing was done on both hindquarters^4,34^. Deficits in the right (uninjected) extremity served as a metric of systemic drug distribution.

Sensory nerve blockade was assessed by modified hotplate testing. In brief, hind paws were exposed in sequence (left then right) to a 56 °C hot plate (Stoelting, Wood Dale, IL, USA), and the time the animal allowed its paw to remain on the hotplate (thermal latency) was measured. A thermal latency of 2 s indicated no nerve blockade (baseline), and a thermal latency of 12 s was maximal latency. Successful nerve blockade was defined as achieving a thermal latency above 7 s. Hind paws were removed from the hotplate after 12 s to prevent thermal injury. Measurements were repeated three times in each animal at each time point and the median was used for further data analysis.

Motor nerve block was assessed by a weight-bearing test to determine the motor strength of the rat’s hindpaw. In brief, the rat was positioned with one hindpaw on a digital balance and was allowed to bear its own weight. The maximum weight that the rat could bear without the ankle touching the balance was recorded, and motor block was considered achieved when the motor strength was less than half-maximal^4,9^. Measurements were repeated three times at each time point and the median was used for further data analysis.

Durations of sensory block were calculated as the time required for thermal latency to return to 7 s (half way between the baseline and maximal latencies). The duration of motor block was defined as the time it took for the weight bearing to return to halfway between normal and maximal block.

### Confocal imaging

Under brief isoflurane-oxygen anesthesia, rats were injected with 0.5 mL of test formulation (25 mg of FITC-T_g_D_8_ conjugates in PEG200, 0.25 mg of fluorescein sodium in PEG200, 0.25 mg of fluorescein sodium in PBS), then euthanized at predetermined intervals. Sciatic nerves together with surrounding tissues were harvested and embedded into OCT compound (VWR, Radnor, PA, USA), then frozen and stored at −20 °C. Sections (10 μm) were prepared using a cryostat microtome (Leica CM3050 S, Wetzlar, Germany) and mounted onto glass slides. Afterwards, slides were fixed with pre-cooled 4% paraformaldehyde for 20 min at room temperature, washed in PBS (pH 7.4) 3 times. Finally, slides were mounted with ProLong Gold Antifade Mountant (with 4′,6-diamidino-2-phenylindole, DAPI) and coverslips. All imaging was performed using a Zeiss LSM 710 multi-photon confocal microscopy (Carl Zeiss AG, Oberkochen, Germany).

### In vivo imaging system (IVIS) imaging

Under isoflurane-oxygen anesthesia, rats were shaved and injected with 0.5 mL of test formulation (25 mg of Cy5.5-T_g_D_8_ conjugates in PEG200). The in vivo fluorescence images were captured and the fluorescence intensity was evaluated at predetermined time points post-injection (under brief isoflurane-oxygen anesthesia) using a Spectrum IVIS (PerkinElmer, Waltham, MA, USA). Whole body animal images were recorded non-invasively. The 675 nm excitation filter and the 700 nm emission filter were used for the imaging. For ex vivo tissue distribution studies, rats were euthanized 1 day after the injection, and the sciatic nerve and surrounding tissues were imaged.

### Tissue harvesting and histology

Rats were sacrificed at 4 and 14 days after the injection (as we have found that these time points are useful in evaluating both acute and chronic inflammation and myotoxicity), and the sciatic nerve was harvested together with surrounding tissues. The dissector was blinded to which solution each rat had been injected with.

Muscle samples were fixed in 10% neutral buffered formalin and processed for histology (hematoxylin–eosin stained slides) using standard techniques. Slides were analyzed by an observer (MM) blinded to the nature of individual samples. Specimens were scored for inflammation (0–4 points) and myotoxicity (0–6 points)^21,35^. The inflammation score was a subjective assessment of severity (0: no inflammation, 1: peripheral inflammation, 2: deep inflammation, 3: muscular hemifascicular inflammation, 4: muscular holofascicular inflammation). The myotoxicity score reflected two characteristic features of local anesthetic myotoxicity: nuclear internalization and regeneration. Nuclear internalization is characterized by myocytes normal in size and chromicity, but with nuclei located away from their usual location at the periphery of the cell^9^. Regeneration is characterized by shrunken myocytes: cells with scant eosinophilic cytoplasm and hyperchromatic nuclei. Scoring was as follows: 0. normal; 1. perifascicular internalization; 2. deep internalization (>5 cell layers), 3. perifascicular regeneration, 4. deep regeneration (>5 cell layers), 5. hemifascicular regeneration, 6. holofascicular regeneration. The grade for a sample represents the worst area (most severe damage) present on the slide.

The sciatic nerves were fixed in Karnovsky’s KII solution, processed and Epon-embedded for toluidine blue staining. They were assessed by optical microscopy in a masked fashion.

### Statistics

Data are presented as means ± SDs (*n* = 4 in release kinetics, cell work, neurobehavioral, and histology studies). The statistical differences between groups were tested by one-way analysis of variance (ANOVA) for multiple comparisons using Origin software (OriginLab Corp. Northampton, MA, USA). *p* < 0.05 was considered to denote statistical significance.

### Statement of ethical compliance

Healthy adult male Sprague–Dawley rats weighting 350–400 g were purchased from Charles River Laboratories and care for in accordance with protocols approved institutionally and nationally. Experiments were carried out in accordance with the Boston Children’s Hospital Animal Use Guidelines and approved by Boston Children’s Hospital’s Animal Care and Use Committee.

## Supplementary information

Supplementary Information

Supplementary Movie 1

## Data Availability

The data that support the findings of this study are available within the paper and its Supplementary Information or from the corresponding authors upon reasonable request.
