# Supplementary material for: Polymer-tetrodotoxin conjugates to induce prolonged duration local anesthesia with minimal toxicity
Source: Nat Commun. 2019 Jun 12;10:2566. doi: 10.1038/s41467-019-10296-9 (PMC6561913; doi:10.1038/s41467-019-10296-9)
Supplement: Supplementary file 1 — Supplementary Information [file 41467_2019_10296_MOESM1_ESM.pdf]

Supplementary Information

Polymer-Tetrodotoxin Formulation to Induce Prolonged Duration Local Anesthesia with Minimal Toxicity

Zhao et al.

Supplementary Tables

Supplementary Table 1. Synthesis and characterization of TDP polymers

Polymer	Feed Dicarboxylic acid (mmol)	Feed Triol (mmol)	Feed PEG (mmol)	PEG concentration[a] (%)	Percent yield (%)[b]
$T_gD_8P_{2000}$	5	1.25	2.5	68.4	88
$T_gD_8P_{1000}$	5	1.25	2.5	60.8	90
$T_gD_8P_{200}$	10	2.5	5	26.7	94
T_gD_8	10	5.8	-	-	96
T_gD_5	10	5.8	-	-	94
T_gD_1	10	5.8	-	-	90
T_cD_8	10	5.8	-	-	95

[a] PEG concentration was calculated according to the NMR ratio of methylene hydrogens within PEG and sebacic acid.

[b]After reaction, the reaction mixture was washed two times with 40 mL of DI water. Polymers were collected after centrifugation at 48,384 xg for 5 min, followed by freezing with liquid nitrogen and lyophilization. Actual yield of polymers was calculated by weighing the dried polymers and all reactants. Percent yield (%) of polymers was calculated as follows:

$$\%\ \text{Yield} = \frac{\text{Actual Yield}}{\text{Theoretical Yield}} \times 100\% \quad (1)$$

Supplementary Table 2. TTX solubility in organic solvents

Organic solvent	TTX solubility (µg mL^{-1})
DMSO	10.0
DMF	1.7
DCM	0

Please see Methods for description of the equilibrium solubility method to determine solubility.

Supplementary Table 3. An example of measurement of the proportion of TTX bound to TD and TDP polymers (vs. unbound) to the total TTX added to the reaction

	Added DMSO (mL)	Added water (mL)	Measured TTX concentration (ng mL^{-1})[b]	Calculated Unbound TTX (μg)
Supernatant #1[a]	10	30	150	6
Supernatant #2[a]	0	40	0	0

Please see Methods for description of the measurement of proportion of drug binding

[a]After Steglich esterification reaction, DCM in the reaction mixture was removed by rotary evaporation, then the reaction mixture (10 mL of DMSO containing polymer-TTX conjugate and unbound TTX) was washed with 30 mL of DI water. The reaction mixture was centrifuged at 48,384 xg for 5 min, and the supernatant (30 mL of DI water, 10 mL of DMSO and unbound TTX) was collected as supernatant #1. The pellet (polymer-TTX conjugates) were washed with 40 mL of DI water and centrifuged again. The supernatant (40 mL of DI water and unbound TTX) was collected as supernatant #2.

[b]The TTX concentration of supernatants was measured with a TTX ELISA kit. To avoid and control for potential interactions of the organic solvents on the ELISA measurements, supernatant #1 was diluted in PBS to a final concentration of 10 % DMSO, and the standard curve of ELISA was generated by dissolving free TTX in a PBS solution containing 10 % DMSO.

In the example above, the proportion of TTX bound to polymer was calculated as follows:

$$\frac{\mathrm{TTX_{feed}} - \mathrm{TTX_{unbound}}}{\mathrm{TTX_{feed}}} \times 100\% = \frac{1000\ \mu g - 6\ \mu g}{1000\ \mu g} \times 100\% = 99.4\% \qquad (2)$$

Supplementary Table 4. Molecular weight of T_gD_8, T_gD_8-TTX and T_gD_8-TTX_H

Polymer	Mn[a]	Mw[a]	PDI[a]
T_gD_8	6011	16564	2.756
T_gD_8-TTX	6107	15780	2.584
T_gD_8-TTX_H	5955	15346	2.577

[a] Molecular weight of polymers were measured by GPC

Supplementary Table 5. Synthesis and characterization of TDP-Dexamethasone conjugates

Conjugate	Feed Dicarboxylic acid (mmol)	Feed Triol (mmol)	Feed PEG (mmol)	Feed Dex[a] (mmol)	Dex loading[b] (µg mg^{-1})	Proportion of bound Dex[c] (%)
$T_gD_8P_{2000}$-Dex	5	1.25	2.5	0.026	1.6	99.1
$T_gD_8P_{1000}$-Dex	5	1.25	2.5	0.026	2.8	99.0
$T_gD_8P_{200}$-Dex	10	2.5	5	0.026	3.1	99.4
T_gD_8-Dex	10	5.8	-	0.026	4.0	99.4
T_gD_5-Dex	10	5.8	-	0.026	5.4	99.5
T_gD_1-Dex	10	5.8	-	0.026	6.4	99.5
T_cD_8-Dex	10	5.8	-	0.026	2.7	99.6

[a] Dex = dexamethasone.

[b] µg dexamethasone per mg polymer, as determined by HPLC.

[c] Proportion of dexamethasone conjugated with polymer as a fraction of the total TTX in the reaction.

Supplementary Table 6. Effect of medium on nerve block induced by free TTX (n = 4).

Function	3 µg of TTX[a]	3 µg of TTX+32 mM SOS[a]	3 µg of TTX[b]	3 µg of TTX+25 mg of T_gD_8[b]	4 µg of TTX+25 mg of T_gD_8[b]	25 mg of T_gD_8[b]
.Successful blocks (%)	25	100	100	100	100	0
Block duration (hours)[c]	0.3±0.5	6.3±0.9	5.3±0.3	3.5±1.1	-	0
Contralateral block (%)	25	100	0	50	100	0
Mortality (%)	0	0	0	0	100	0

[a] Injected with 0.5 mL of PBS

[b] Injected with 0.5 mL of PEG200

[c] Data for durations of nerve block are means ± SD.

Supplementary Table 7. Mass of TD-TTX and TDP-TTX conjugates and dose of TTX in 0.5 mL of PEG200 injected in vivo.

Polymer-TTX conjugate	TTX loading (μg mg^{-1})	Mass of Polymer-TTX conjugate (mg)	Dose of TTX (μg)
T_gD_8-TTX	0.4	2.5	1
		5	2
		7.5	3
		10	4
		12.5	5
		25	10
T_gD_8-TTX_H	1.6	12.5	20
		25	40
		50	80
$T_gD_8P_{2000}$-TTX	0.16	6.25	1
		62.5	10
$T_gD_8P_{1000}$-TTX	0.28	3.6	1
		36	10
$T_gD_8P_{200}$-TTX	0.31	3.3	1
		33	10
T_gD_1-TTX	0.64	1.6	1
		16	10

Supplementary Table 8. Effect of free and conjugated TTX in 1:4 PEG200/PBS solution on peripheral nerve blockade. (n = 4)

Function	1 µg of free TTX	40 µg of TTX in T_gD_8-TTX_H
Successful blocks (%)	0	0
Block duration (hours)	0	0
Contralateral block (%)	0	0
Mortality (%)	0	0

Data for durations of nerve block are means ± SD.

Supplementary Table 9. Toxicity of conjugated TTX with intravenous injection. (n = 4)

Function	20 μg of TTX in T_gD_8-TTX_H[a]
Sensory deficits (%)	0
Motor deficits (%)	0
Mortality (%)	0

[a] T_gD_8-TTX_H/PEG200 solution (50 mg mL^{-1}) was diluted with PBS containing 10 wt% BSA by 1:1 to a final concentration of 25 mg mL^{-1}.

Supplementary Figures

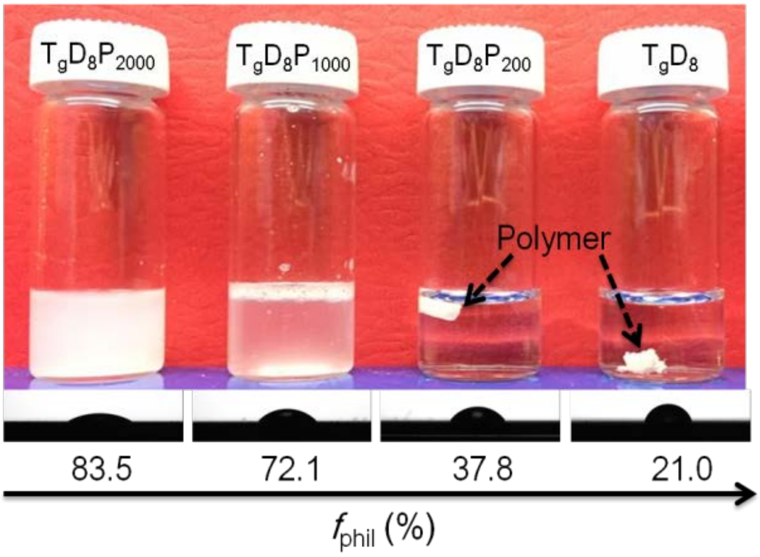

Supplementary Figure 1. Suspension of TD and TDP polymers in PBS.

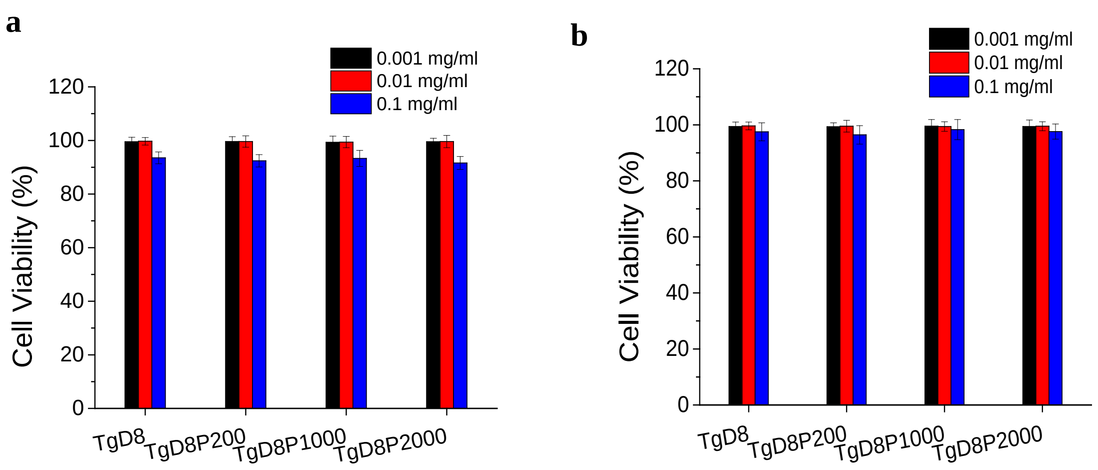

Supplementary Figure 2. Cytotoxicity of cells incubated with various concentration of TDP polymers. TDP polymers were added to the cell culture and cell viability was evaluated by the MTT assay after 24 h. a). Viability of C2C12 cells. b). Viability of PC12 cells. Data were normalized so that 100% represents viability in untreated cells. Data are means ± SD, n=4.

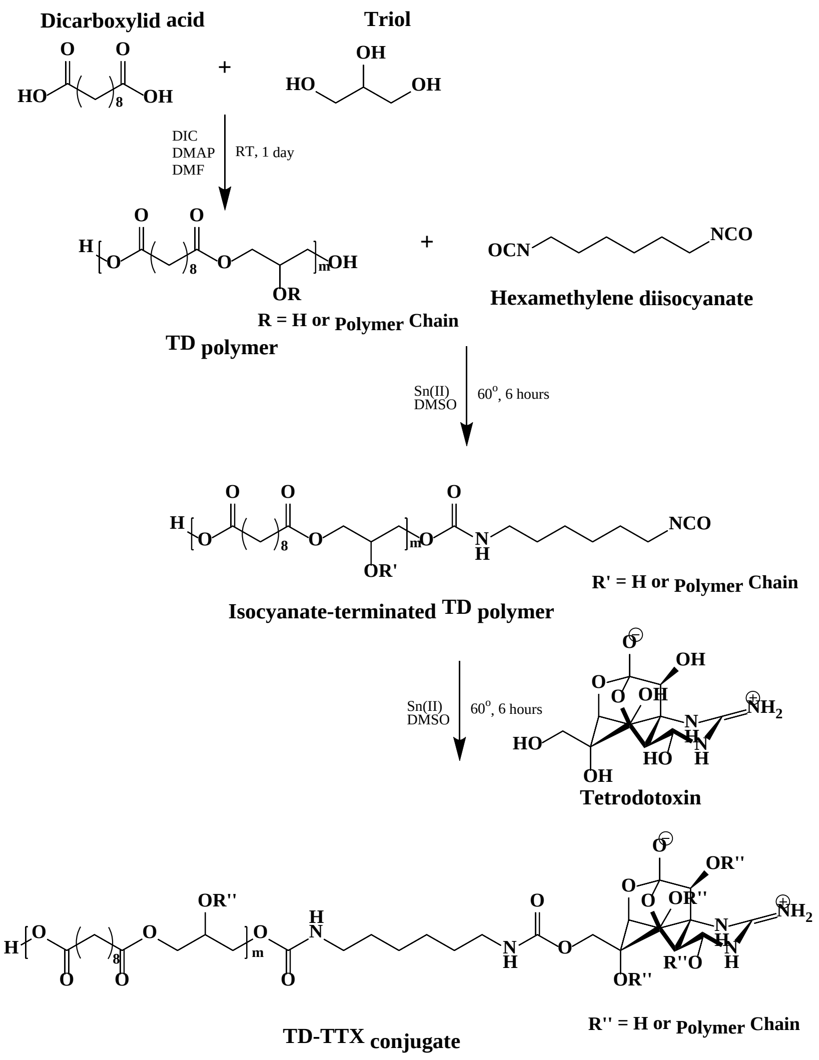

Supplementary Figure 3. Synthesis of T_gD_8-TTX urethane conjugates.

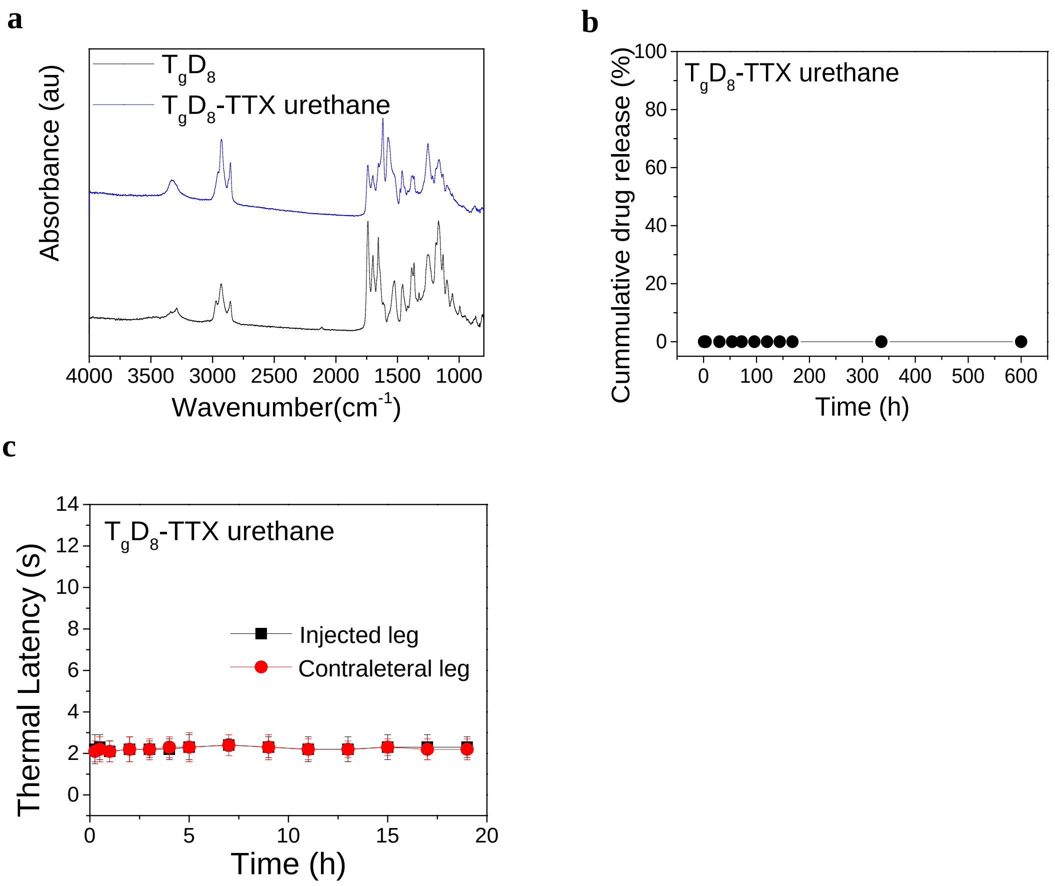

Supplementary Figure 4. Characterization of T_gD_8-TTX urethane conjugates. a) FTIR spectra of T_gD_8 polymer and T_gD_8-TTX urethane conjugates. b) TTX release from 25 mg of T_gD_8-TTX urethane conjugate as measured by ELISA. c) Representative sciatic nerve block in a single rat injected with 25 mg of T_gD_8-TTX urethane conjugate containing 10.0 µg of TTX formulated in 0.5 mL of PEG200. Data are means ± SD, measurements were repeated three times at each time point.

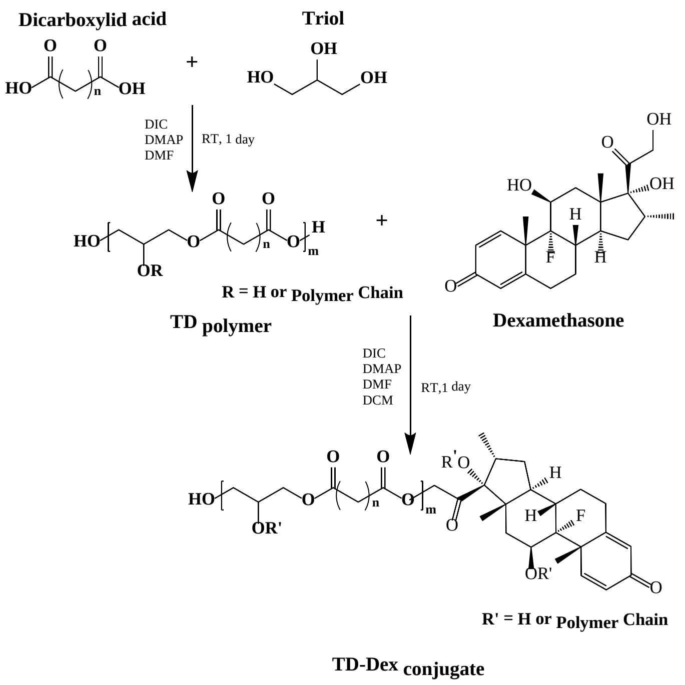

Supplementary Figure 5. Steglich esterification reaction for synthesis of TD-Dex conjugates. Dex=dexamethasone.

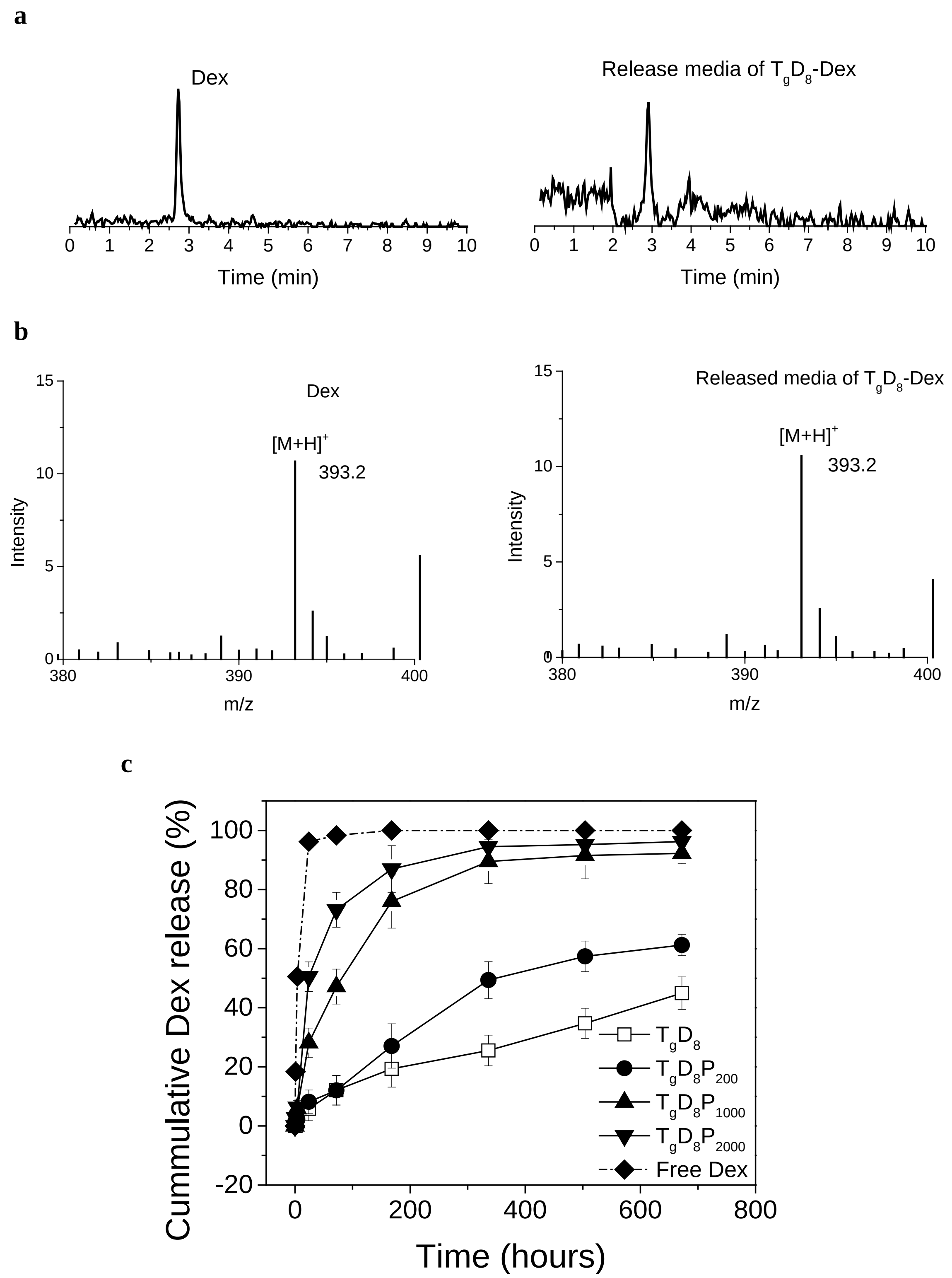

Supplementary Figure 6. In vitro release of dexamethasone from TDP-Dex conjugates. (a) HPLC analyses of pure dexamethasone and release media of TDP-Dex conjugates showed single peak at ~ 2.9 min. (b) LC-MS confirmed that the molecular weight of the compound (m/z 393 is $[M+H]^+$) observed at ~ 2.9 min in HPLC corresponded to that of pure dexamethasone (left panel). c) Release profile of 100 µg of free dexamethasone or

dexamethasone from TDP-Dex conjugates with differing hydrophilic fraction. In panel (c), the white boxes denote T_gD_8. Dex= dexamethasone. Data are means ± SD, n=4.

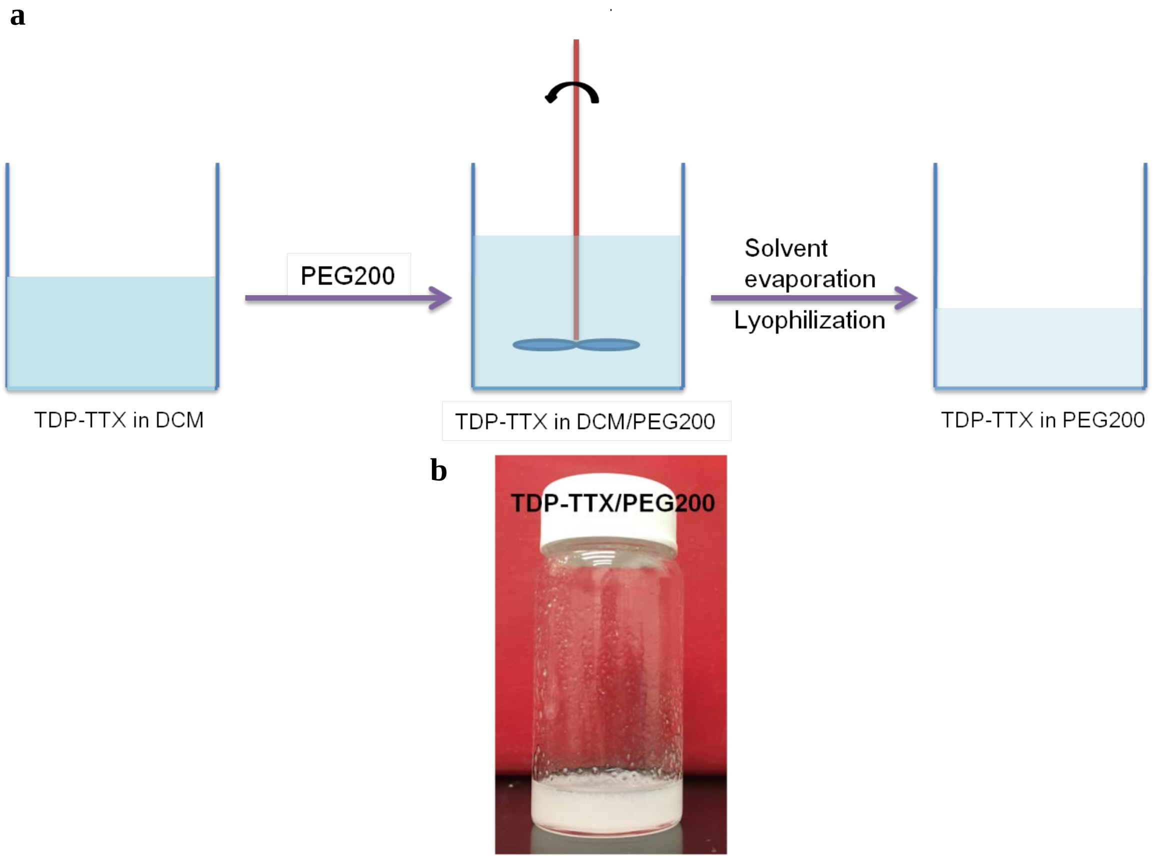

Supplementary Figure 7. Fabrication of injectable TDP-TTX/PEG200 formulation. a) General scheme of solvent evaporation method. Please see Methods for further details. b) A homogeneous suspension of T_gD_8-TTX conjugate in PEG200 (50 mg mL^{-1}).

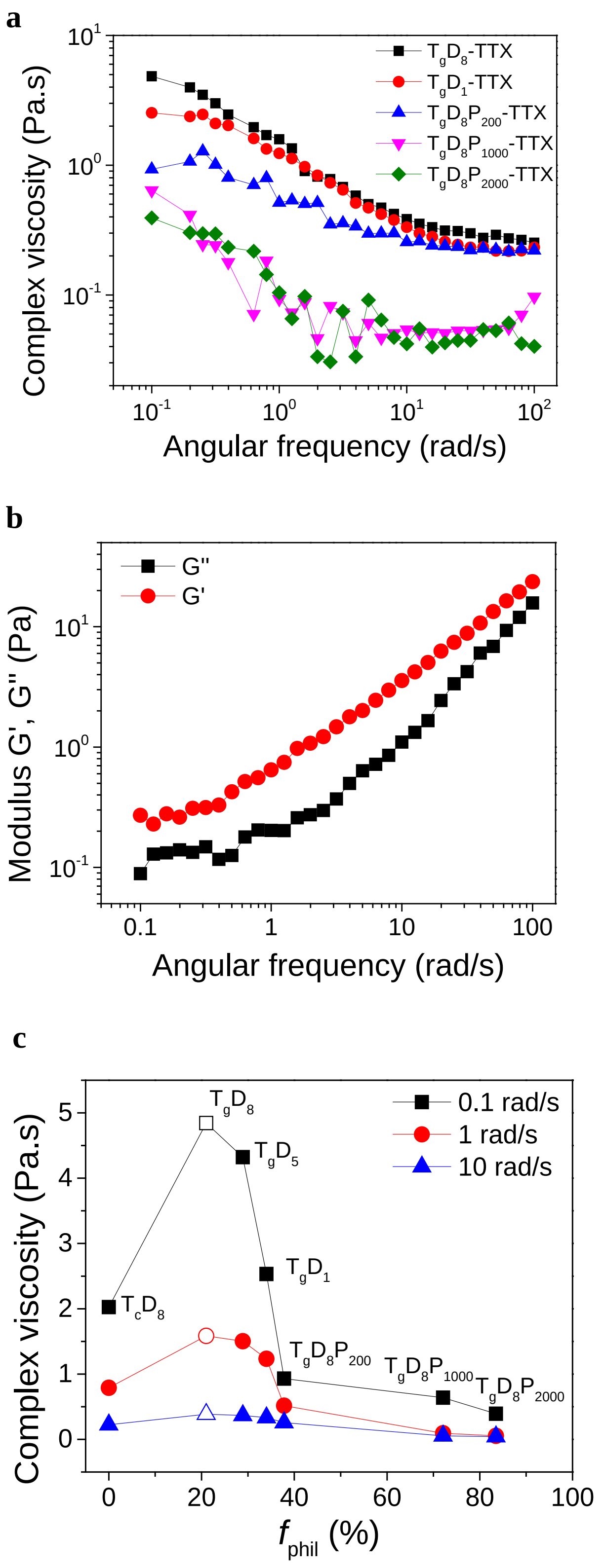

Supplementary Figure 8. Viscoelastic properties of the formulations. a) Complex viscosity of formulations of TD-TTX/PEG200 or TDP-TTX/PEG200 (50 mg mL^{-1}). b) Moduli of T_gD_8-TTX/PEG200 (50 mg mL^{-1}). c) Effect of hydrophilic fraction of the polymers (f_{phil}) in PEG200 on the viscosity of formulations at 0.1, 1, and 10 rad/s. In panel (c), the whites box denote T_gD_8. n=4 per experimental group.

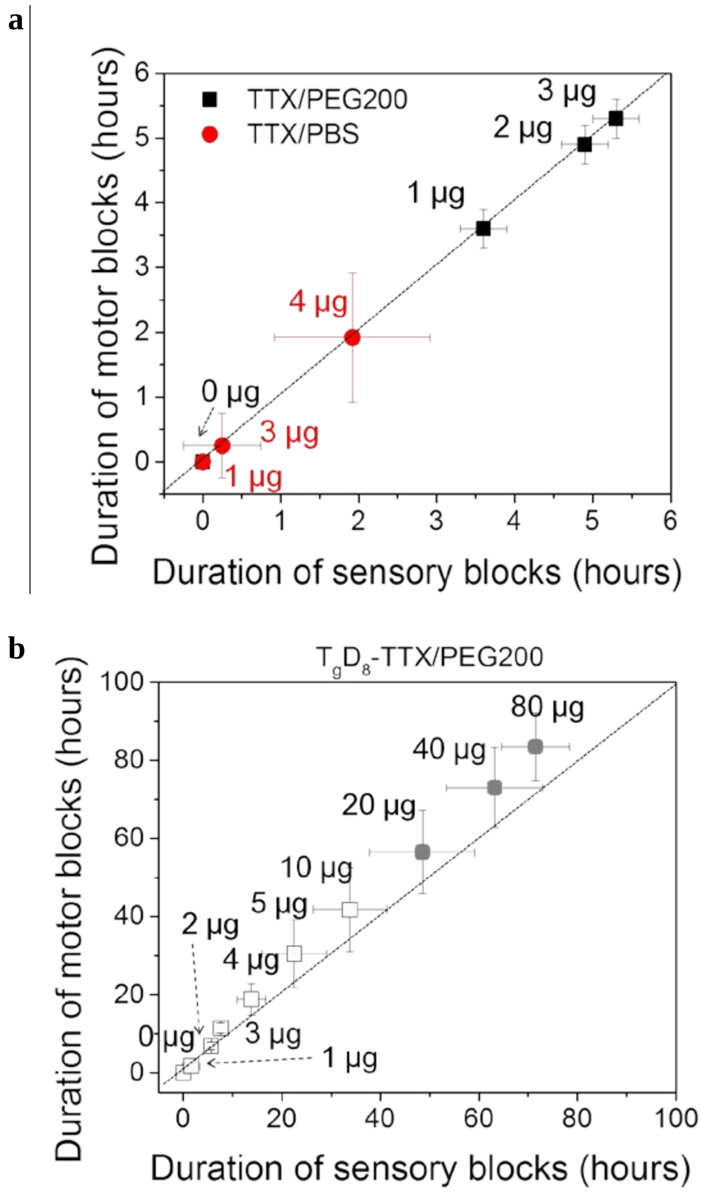

Supplementary Figure 9. Comparison of the durations of sensory and motor blocks. Animals were injected with a) free TTX in 0.5 mL of PBS or PEG200, b) T_gD_8-TTX or T_gD_8-TTX_H in 0.5 mL of PEG200. In panel (b), the white boxes denote T_gD_8-TTX, grey boxes denote T_gD_8-TTX_H. Data are means ± SD, n=4.

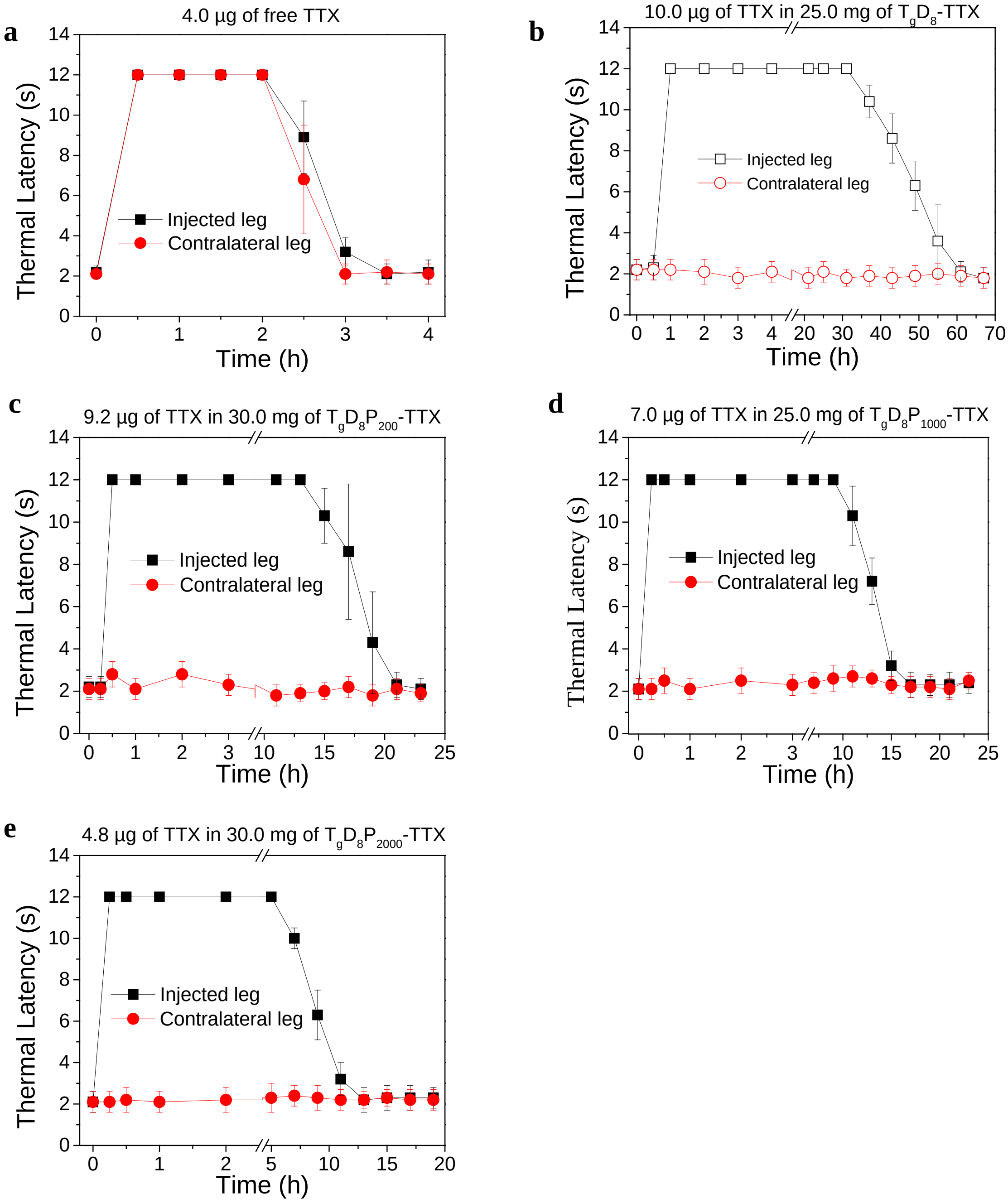

Supplementary Figure 10. Comparison of efficacy and systemic toxicity from selected formulations. Thermal latency in the injected extremity assesses efficacy, latency in the contralateral (uninjected) extremity assesses systemic toxicity. Panels are representative time courses of sciatic nerve blocks in single rats injected with a) 4 µg of free TTX, b) 25 mg of T_gD_8-TTX conjugate containing 10.0 µg TTX, c) 30 mg of $T_gD_8P_{200}$-TTX conjugate containing 9.2 µg of TTX, d) 25 mg of $T_gD_8P_{1000}$-TTX conjugate containing 7.0 µg TTX e) 30 mg of $T_gD_8P_{2000}$-TTX conjugate containing 4.8 µg TTX. In panel (b), the white boxes denote T_gD_8-TTX. Data are means ± SD, measurements were repeated three times at each time point. Data are representative of 4 animals in each group.

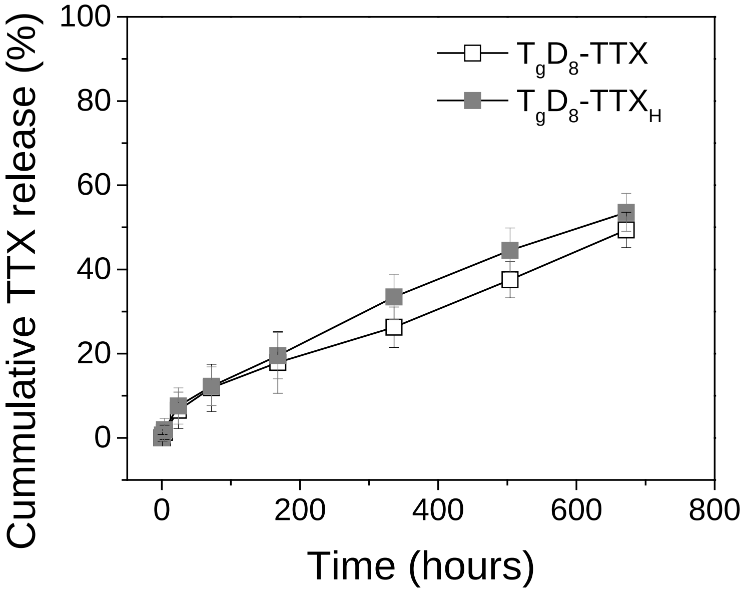

Supplementary Figure 11. Release profile of 10 µg of TTX from T_gD_8-TTX and T_gD_8-TTX_H conjugates. The white boxes denote T_gD_8-TTX, grey boxes denote T_gD_8-TTX_H. Data are means ± SD, n = 4.

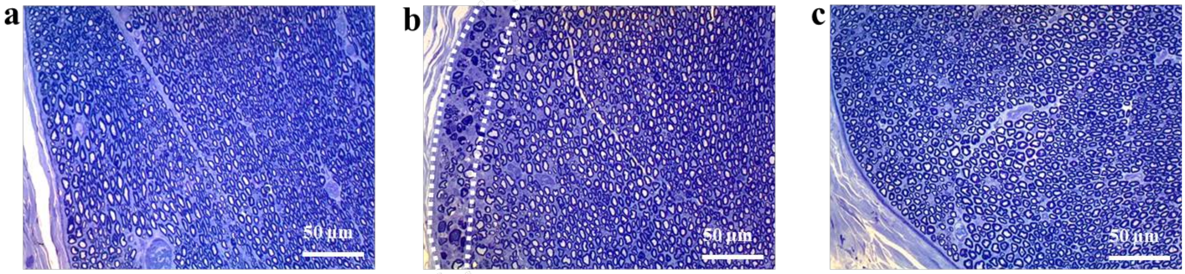

Supplementary Figure 12. Representative toluidine blue stained sections of sciatic nerves. (a) uninjected nerve, (b) nerve 4 days after injection of PEG200 (the area enclosed by white dotted lines indicates minimal peripheral injury) and (c) nerve 4 days after injection of PPG4000. Data are representative of 3 animals in each group. The scale bar is 50 µm.

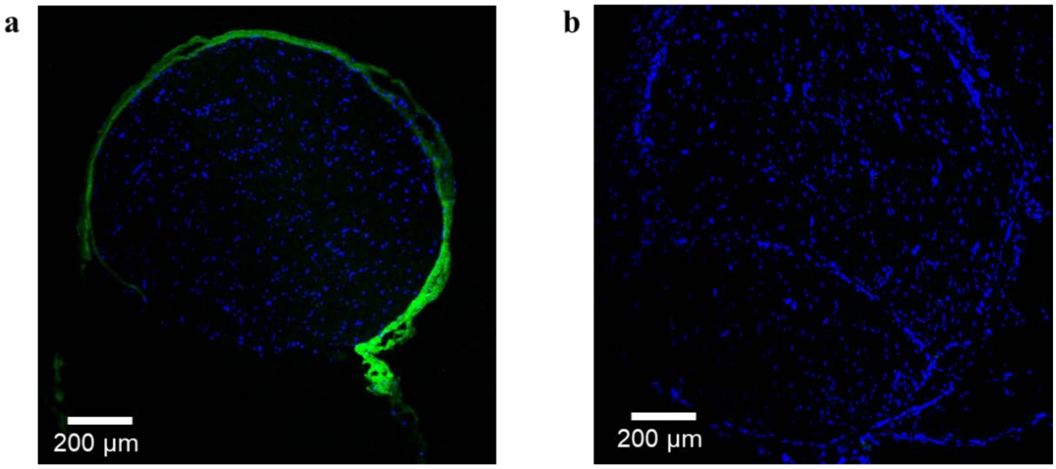

Supplementary Figure 13. Representative fluorescence microscopy images of sections of sciatic nerves and surrounding tissues. (a) 1 hour after injection of 0.25 mg of fluorescein sodium in 0.5 mL of PPG4000, (b) without injection, on the contralateral side. Green: fluorescein sodium. Blue: cell nuclei (stained with DAPI). Data are representative of 4 animals in each group. The scale bar is 200 µm.

Supplementary Notes

Supplementary Note 1:

To demonstrate the importance of covalent bonding to the nerve blockade and toxicity of these formulations, free TTX was physically mixed with 25 mg of T_gD_8 polymer in 0.5 mL of PEG200 and injected at the sciatic nerve. Block from 3 µg (18 µM) of TTX was successful in 100% of animals and produced a median duration of sensory nerve block of 3.5 ± 1.1 hours. However, the blocks were associated with marked systemic toxicity, as evidenced by sensory deficits in the uninjected (contralateral) leg (Supplementary Table 6). 4 µg (24 µM) of free TTX caused contralateral deficits in all animals and was subsequently uniformly fatal (Supplementary Table 6). These results indicated that the covalent bonding of TTX to T_gD_8 had a marked impact on nerve block and safety.

Supplementary Note 2:

To determine whether both PEG200 and T_gD_8 were responsible for CPE-like effects, 3 µg (18 µM) of free TTX together with (but not conjugated to) 25 mg of T_gD_8 polymer in 0.5 mL of PEG200 were injected at the sciatic nerve, resulting in 100% blockade with a duration of 3.5±1.1 h. This was longer than the duration of block from free TTX (p = 0.002, one-way ANOVA), but not than that from TTX in PEG200 (p = 0.020, one-way ANOVA), suggesting that the PEG200 and not the T_gD_8 was responsible for the CPE-like effect. In the absence of TTX, T_gD_8/PEG200 did not cause nerve block (Supplementary Table 6).

Supplementary Note 3:

We investigated the relative contributions to nerve block of the CPE effect of PEG200 and of the sustained release of TTX from T_gD_8-TTX_H by formulating the polymer in an injectate with diluted PEG200. We diluted the T_gD_8-TTX_H/PEG200 solution (200 mg mL^{-1}) 1:4 in PBS containing 10 wt% bovine serum albumin (BSA), to a final concentration of 50 $mgmL^{-1}$. (BSA was used to solubilize the T_gD_8-TTX as it is known to bind hydrophobic molecules[1,2].) When injected at the sciatic nerve, 25 mg of T_gD_8-TTX_H containing 40 µg (240 µM) of TTX in 0.5 mL of the diluted solution (1:4 PEG200/BSA) did not cause detectable nerve block or toxicity; when delivered in undiluted PEG200, T_gD_8-TTX_H containing 40 µg (240 µM) of TTX had caused nerve block lasting 63.2±9.8 hours (Fig. 5b). The lack of CPE effect at the concentration of PEG in 1:4 PEG/BSA was demonstrated by the fact that 1 µg (6 µM) of free TTX in 0.5 mL of 1:4 PEG/BSA caused no detectable nerve block or toxicity (Supplementary Table 8), whereas the same concentration of TTX in undiluted PEG200 yielded a nerve block lasting 3.6±0.3 hours (Fig. 5b). Consequently, the prolonged nerve block from T_gD_8-TTX_H/PEG200 was dependent on both the sustained release of TTX from T_gD_8-TTX_H and the CPE effect of PEG200.

Supplementary Discussion

Supplementary Discussion 1:

Polymer-TTX conjugates have the potential to produce even longer nerve block durations than those achieved in this work. In vitro, sustained release of TTX from T_gD_8-TTX conjugates followed a near linear profile for over one month. However, in vivo, the T_gD_8-TTX/PEG200 formulation produced durations of nerve block no longer than 3 days when the TTX dose increased from 40 to 80 μg ($p > 0.05$, one-way ANOVA). The reason increasing the dose further did not increase duration can be that the material is not retained in tissue for more than 4 days (Fig. 7a). Longer nerve blocks might be achieved by polymers with longer local retention in tissue.

Supplementary Discussion 2:

Here, we demonstrated prolonged duration local anesthesia in peripheral nerve. In principle, these formulations could also be used in the neuraxis. In either location, the effect of the site 1 sodium channel blocker could be enhanced by co-administration of other drugs[3]. For example, the duration of block from site 1 sodium channel blockers can be prolonged by conventional local anesthetics[4,5], vasoconstrictors[4], and glucocorticoid receptor agonists[6,7]. In the neuraxis, conventional local anesthetics' performance can be improved by opioids[8,9], alpha-adrenergic agents[9,10], and other drugs[9]; whether the same would be true for site 1 sodium channel blockers is not known. Formulation such as these could find uses other than in pain. For example, sustained release of tetrodotoxin has been shown to have anticonvulsant effects[11].

Supplementary References

1. Takehara, K., Yuki, K., Shirasawa, M., Yamasaki, S. & Yamada, S. Binding Properties of Hydrophobic Molecules to Human Serum Albumin Studied by Fluorescence Titration. *Anal. Sci.* **25,** 115–120 (2009).
2. Zsila, F. *et al.* Evaluation of drug–human serum albumin binding interactions with support vector machine aided online automated docking. *Bioinformatics* **27,** 1806–1813 (2011).
3. Santamaria, C. M., Woodruff, A., Yang, R. & Kohane, D. S. Drug delivery systems for prolonged duration local anesthesia. *Mater. Today* **20,** 22–31 (2017).
4. Adams, H. J., Blair, M. R. & Takman, B. H. The local anesthetic activity of tetrodotoxin alone and in combination with vasoconstrictors and local anesthetics. *Anesth. Analg.* **55,** 568—573 (1976).
5. Kohane Daniel S., M. D. P. *et al.* A Re-examination of Tetrodotoxin for Prolonged Duration Local Anesthesia. *Anesthesiol. J. Am. Soc. Anesthesiol.* **89,** 119–131 (1998).
6. Kohane, D. S. *et al.* Prolonged duration local anesthesia from tetrodotoxin-enhanced local anesthetic microspheres. *Pain* **104,** 415–421 (2003).
7. Epstein-Barash, H. *et al.* Prolonged duration local anesthesia with minimal toxicity. *Proc. Natl. Acad. Sci.* **106,** 7125–7130 (2009).
8. Moulin, D. E. & Coyle, N. Spinal opioid analgesics and local anesthetics in the management of chronic cancer pain. *J. Pain Symptom Manage.* **1,** 79–86 (1986).
9. Schug, S. A., Saunders, D., Kurowski, I. & Paech, M. J. Neuraxial Drug Administration. *CNS Drugs* **20,** 917–933 (2006).
10. Klamt, J. G., Garcia, L. V., Stocche, R. M. & Meinberg, A. C. Epidural infusion of clonidine or clonidine plus ropivacaine for postoperative analgesia in children undergoing major abdominal surgery. *J. Clin. Anesth.* **15,** 510–514 (2003).
11. Graber, K. D. & Prince, D. A. Tetrodotoxin prevents posttraumatic epileptogenesis in rats. *Ann. Neurol.* **46,** 234–242 (1999).
